# Supplementary material for: The ability to classify patients based on gene-expression data varies by algorithm and performance metric
Source: PLoS Comput Biol. 2022 Mar 11;18(3):e1009926. doi: 10.1371/journal.pcbi.1009926 (PMC8942277; doi:10.1371/journal.pcbi.1009926)
Supplement: S1 Fig — We predicted patient states using gene-expression predictors only (Analysis 1). For each combination of dataset, class variable, and classification algorithm, we calculated the arithmetic mean of area under the receiver operating characteristic curve (AUROC) values across 50 iterations of Monte Carlo cross-validation. Next, we sorted the algorithms based on the average rank across all dataset/class combinations. Each data point that overlays the box plots represents a particular dataset/class combination. The top 15 performers (relatively low ranks) were algorithms that use linear decision boundaries, kernel functions, and/or ensembles of decision trees. (PDF) [file pcbi.1009926.s001.pdf]

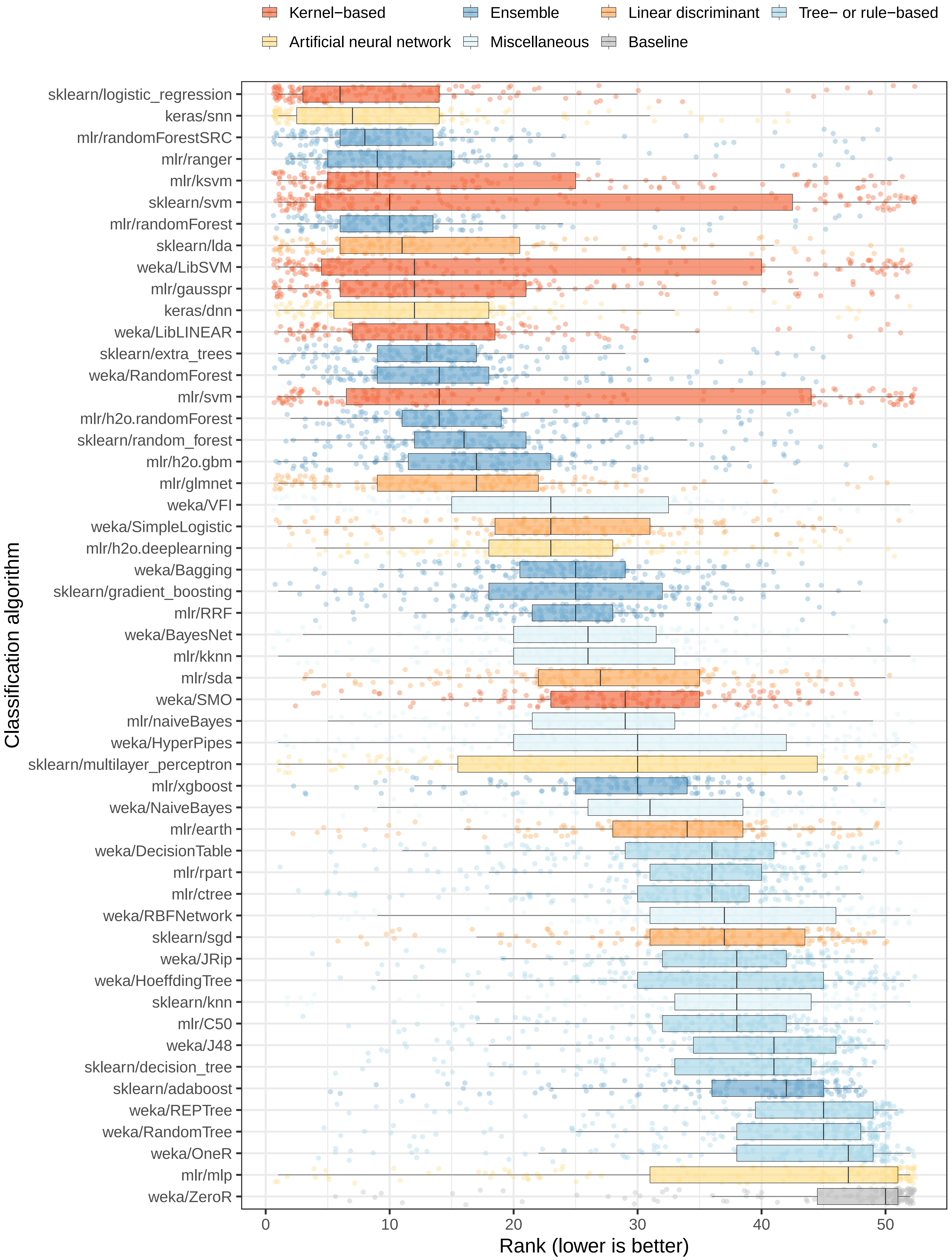
Kernel−based
Ensemble
Linear discriminant
Tree− or rule−based
Artificial neural network
Miscellaneous
Baseline
Classification algorithm
sklearn/logistic_regression
keras/snn
mlr/randomForestSRC
mlr/ranger
mlr/ksvm
sklearn/svm
mlr/randomForest
sklearn/lda
weka/LibSVM
mlr/gausspr
keras/dnn
weka/LibLINEAR
sklearn/extra_trees
weka/RandomForest
mlr/svm
mlr/h2o.randomForest
sklearn/random_forest
mlr/h2o.gbm
mlr/glmnet
weka/VFI
weka/SimpleLogistic
mlr/h2o.deeplearning
weka/Bagging
sklearn/gradient_boosting
mlr/RRF
weka/BayesNet
mlr/kknn
mlr/sda
weka/SMO
mlr/naiveBayes
weka/HyperPipes
sklearn/multilayer_perceptron
mlr/xgboost
weka/NaiveBayes
mlr/earth
weka/DecisionTable
mlr/rpart
mlr/ctree
weka/RBFNetwork
sklearn/sgd
weka/JRip
weka/HoeffdingTree
sklearn/knn
mlr/C50
weka/J48
sklearn/decision_tree
sklearn/adaboost
weka/REPTree
weka/RandomTree
weka/OneR
mlr/mlp
weka/ZeroR
0
10
20
30
40
50
Rank (lower is better)
