# Supplementary material for: The ability to classify patients based on gene-expression data varies by algorithm and performance metric
Source: PLoS Comput Biol. 2022 Mar 11;18(3):e1009926. doi: 10.1371/journal.pcbi.1009926 (PMC8942277; doi:10.1371/journal.pcbi.1009926)
Supplement: S2 Fig — We predicted patient states using gene-expression predictors only (Analysis 1). For each combination of dataset, class variable, and classification algorithm, we calculated the arithmetic mean of classification accuracy across 50 iterations of Monte Carlo cross-validation. Next, we sorted the algorithms based on the average rank across all dataset/class combinations. Each data point that overlays the box plots represents a particular dataset/class combination. (PDF) [file pcbi.1009926.s002.pdf]

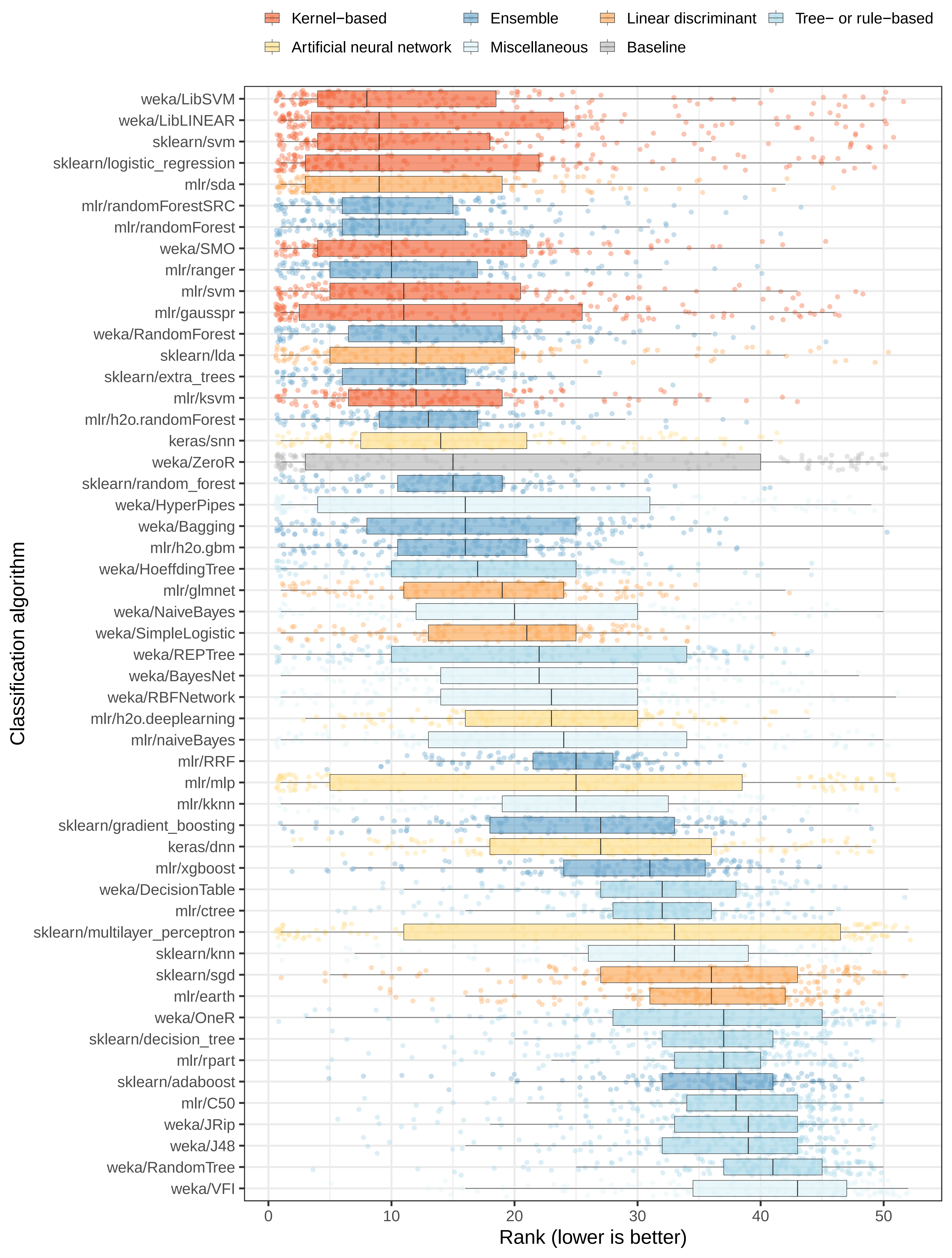
Kernel−based
Ensemble
Linear discriminant
Tree− or rule−based
Artificial neural network
Miscellaneous
Baseline
Classification algorithm
weka/LibSVM
weka/LibLINEAR
sklearn/svm
sklearn/logistic_regression
mlr/sda
mlr/randomForestSRC
mlr/randomForest
weka/SMO
mlr/ranger
mlr/svm
mlr/gausspr
weka/RandomForest
sklearn/lda
sklearn/extra_trees
mlr/ksvm
mlr/h2o.randomForest
keras/snn
weka/ZeroR
sklearn/random_forest
weka/HyperPipes
weka/Bagging
mlr/h2o.gbm
weka/HoeffdingTree
mlr/glmnet
weka/NaiveBayes
weka/SimpleLogistic
weka/REPTree
weka/BayesNet
weka/RBFNetwork
mlr/h2o.deeplearning
mlr/naiveBayes
mlr/RRF
mlr/mlp
mlr/kknn
sklearn/gradient_boosting
keras/dnn
mlr/xgboost
weka/DecisionTable
mlr/ctree
sklearn/multilayer_perceptron
sklearn/knn
sklearn/sgd
mlr/earth
weka/OneR
sklearn/decision_tree
mlr/rpart
sklearn/adaboost
mlr/C50
weka/JRip
weka/J48
weka/RandomTree
weka/VFI
0
10
20
30
40
50
Rank (lower is better)
