# Supplementary material for: The ability to classify patients based on gene-expression data varies by algorithm and performance metric
Source: PLoS Comput Biol. 2022 Mar 11;18(3):e1009926. doi: 10.1371/journal.pcbi.1009926 (PMC8942277; doi:10.1371/journal.pcbi.1009926)
Supplement: S3 Fig — We predicted patient states using gene-expression predictors only (Analysis 1). For each combination of dataset, class variable, and classification algorithm, we calculated the arithmetic mean of the Matthews Correlation Coefficient across 50 iterations of Monte Carlo cross-validation. Next, we sorted the algorithms based on the average rank across all dataset/class combinations. Each data point that overlays the box plots represents a particular dataset/class combination. (PDF) [file pcbi.1009926.s003.pdf]

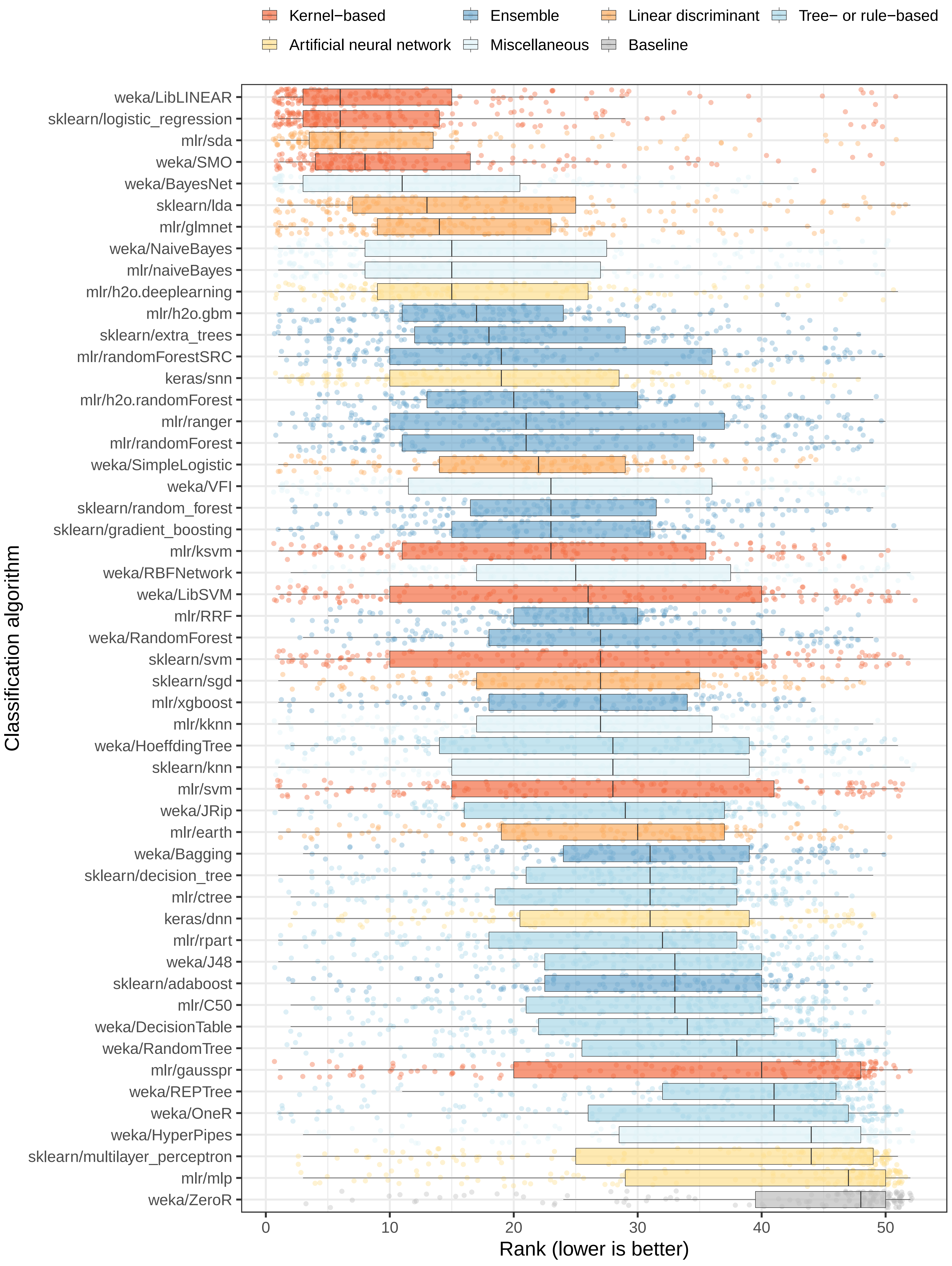
Kernel−based
Ensemble
Linear discriminant
Tree− or rule−based
Artificial neural network
Miscellaneous
Baseline
Classification algorithm
weka/LibLINEAR
sklearn/logistic_regression
mlr/sda
weka/SMO
weka/BayesNet
sklearn/lda
mlr/glmnet
weka/NaiveBayes
mlr/naiveBayes
mlr/h2o.deeplearning
mlr/h2o.gbm
sklearn/extra_trees
mlr/randomForestSRC
keras/snn
mlr/h2o.randomForest
mlr/ranger
mlr/randomForest
weka/SimpleLogistic
weka/VFI
sklearn/random_forest
sklearn/gradient_boosting
mlr/ksvm
weka/RBFNetwork
weka/LibSVM
mlr/RRF
weka/RandomForest
sklearn/svm
sklearn/sgd
mlr/xgboost
mlr/kknn
weka/HoeffdingTree
sklearn/knn
mlr/svm
weka/JRip
mlr/earth
weka/Bagging
sklearn/decision_tree
mlr/ctree
keras/dnn
mlr/rpart
weka/J48
sklearn/adaboost
mlr/C50
weka/DecisionTable
weka/RandomTree
mlr/gausspr
weka/REPTree
weka/OneR
weka/HyperPipes
sklearn/multilayer_perceptron
mlr/mlp
weka/ZeroR
0
10
20
30
40
50
Rank (lower is better)
