# Supplementary material for: The ability to classify patients based on gene-expression data varies by algorithm and performance metric
Source: PLoS Comput Biol. 2022 Mar 11;18(3):e1009926. doi: 10.1371/journal.pcbi.1009926 (PMC8942277; doi:10.1371/journal.pcbi.1009926)
Supplement: S4 Fig — We predicted patient states using gene-expression predictors only (Analysis 1). For each combination of dataset, class variable, and classification algorithm, we calculated the arithmetic mean of area under the precision-recall curve across 50 iterations of Monte Carlo cross-validation. Next, we sorted the algorithms based on the average rank across all dataset/class combinations. Each data point that overlays the box plots represents a particular dataset/class combination. (PDF) [file pcbi.1009926.s004.pdf]

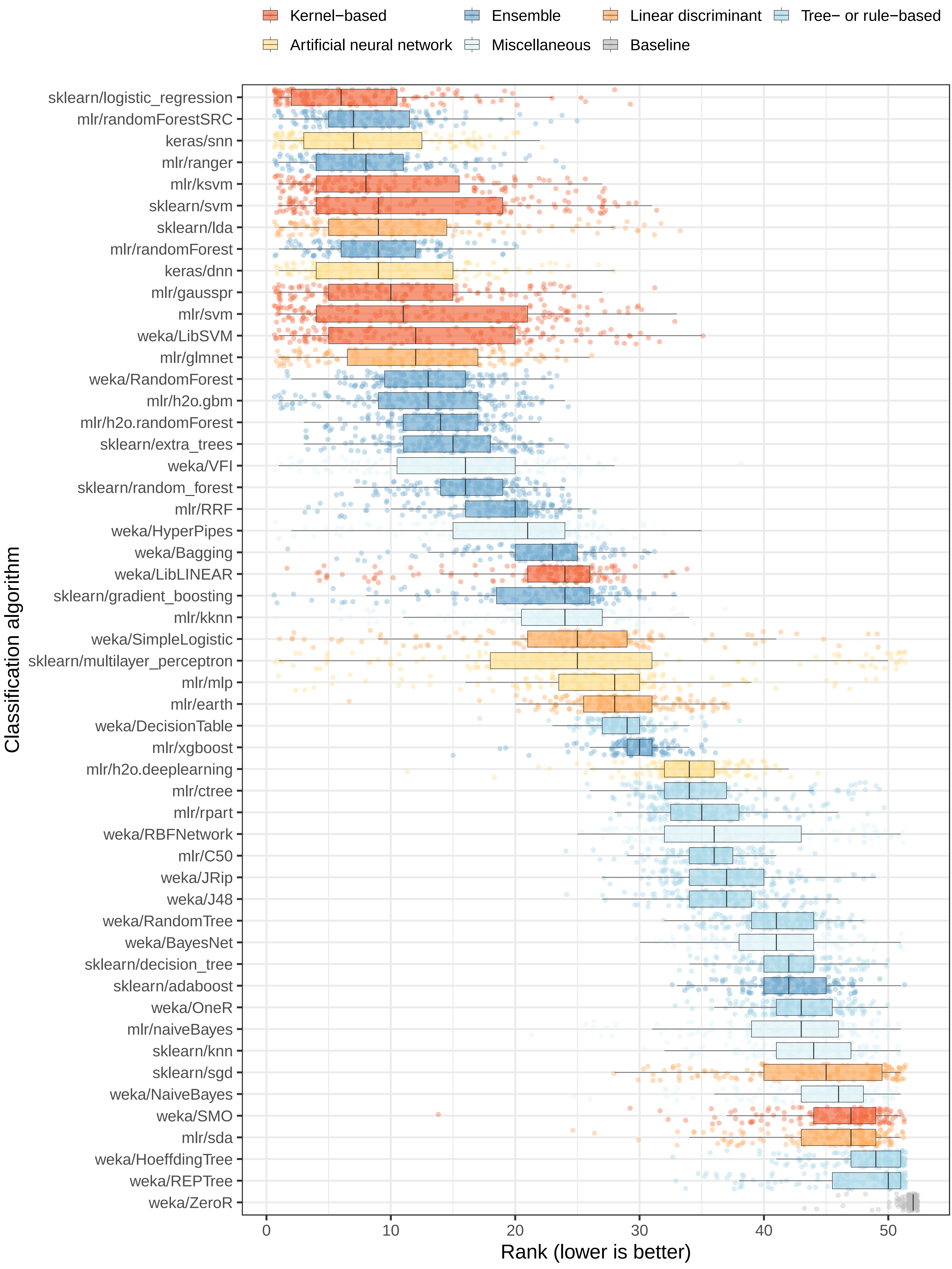
Kernel−based
Ensemble
Linear discriminant
Tree− or rule−based
Artificial neural network
Miscellaneous
Baseline
sklearn/logistic_regression
mlr/randomForestSRC
keras/snn
mlr/ranger
mlr/ksvm
sklearn/svm
sklearn/lda
mlr/randomForest
keras/dnn
mlr/gausspr
mlr/svm
weka/LibSVM
mlr/glmnet
weka/RandomForest
mlr/h2o.gbm
mlr/h2o.randomForest
sklearn/extra_trees
weka/VFI
sklearn/random_forest
mlr/RRF
weka/HyperPipes
weka/Bagging
weka/LibLINEAR
sklearn/gradient_boosting
mlr/kknn
weka/SimpleLogistic
sklearn/multilayer_perceptron
mlr/mlp
mlr/earth
weka/DecisionTable
mlr/xgboost
mlr/h2o.deeplearning
mlr/ctree
mlr/rpart
weka/RBFNetwork
mlr/C50
weka/JRip
weka/J48
weka/RandomTree
weka/BayesNet
sklearn/decision_tree
sklearn/adaboost
weka/OneR
mlr/naiveBayes
sklearn/knn
sklearn/sgd
weka/NaiveBayes
weka/SMO
mlr/sda
weka/HoeffdingTree
weka/REPTree
weka/ZeroR
Classification algorithm
0
10
20
30
40
50
Rank (lower is better)
