# Supplementary figures and images for: The ability to classify patients based on gene-expression data varies by algorithm and performance metric
Source: PLoS Comput Biol. 2022 Mar 11;18(3):e1009926. doi: 10.1371/journal.pcbi.1009926 (PMC8942277; doi:10.1371/journal.pcbi.1009926)

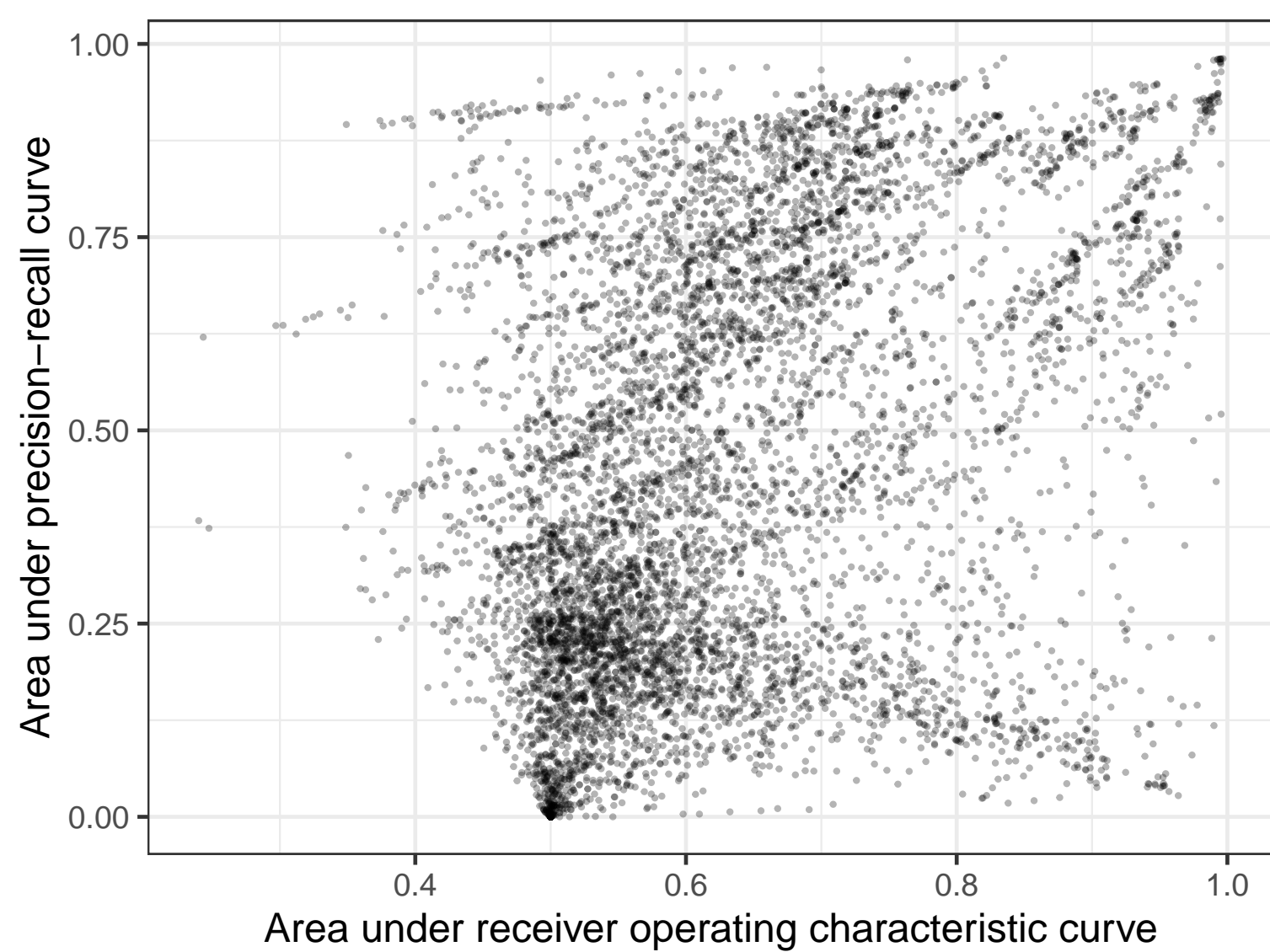

Supplement: S5 Fig — (PDF) [file pcbi.1009926.s005.pdf]

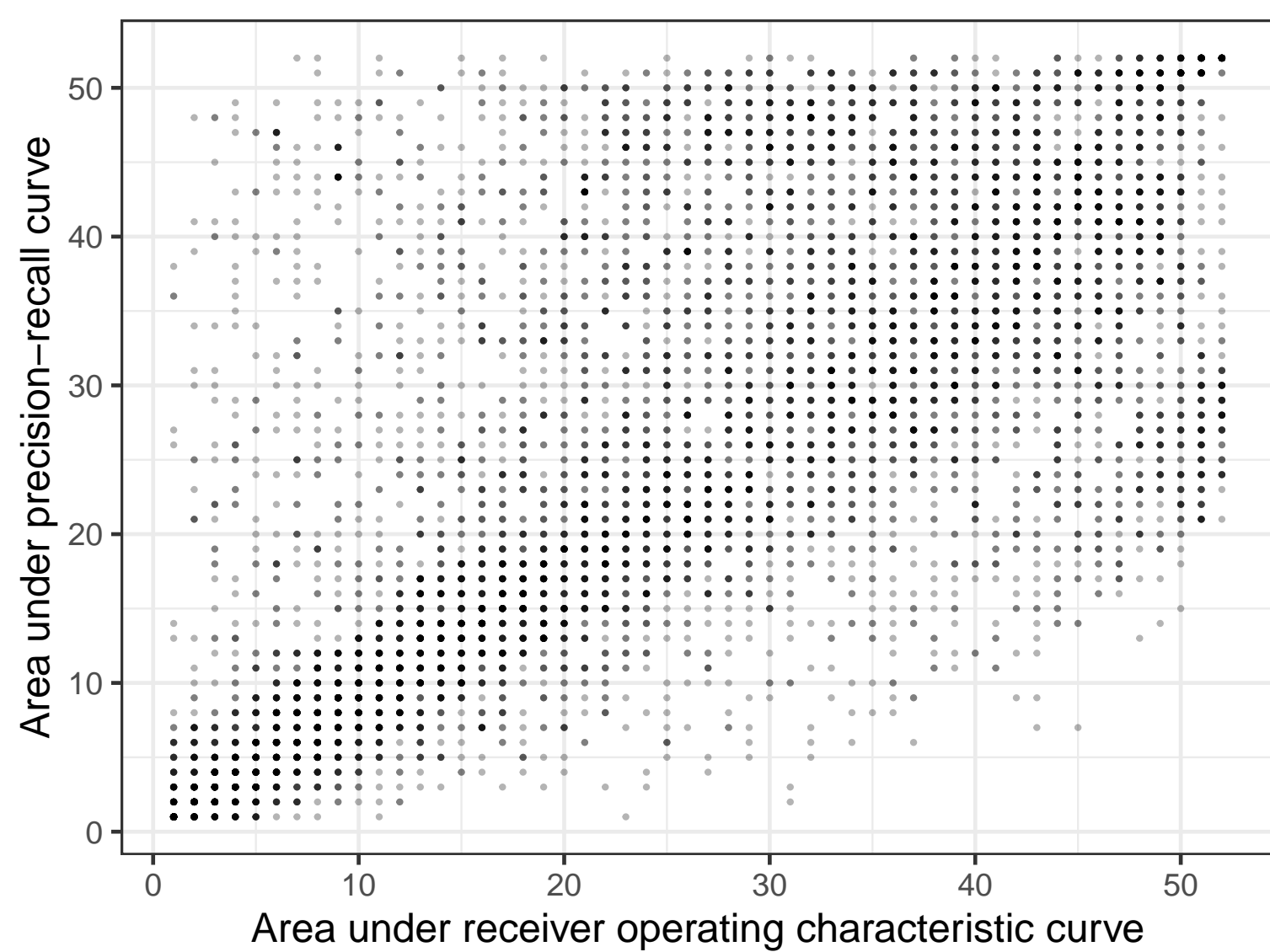

Supplement: S6 Fig — (PDF) [file pcbi.1009926.s006.pdf]

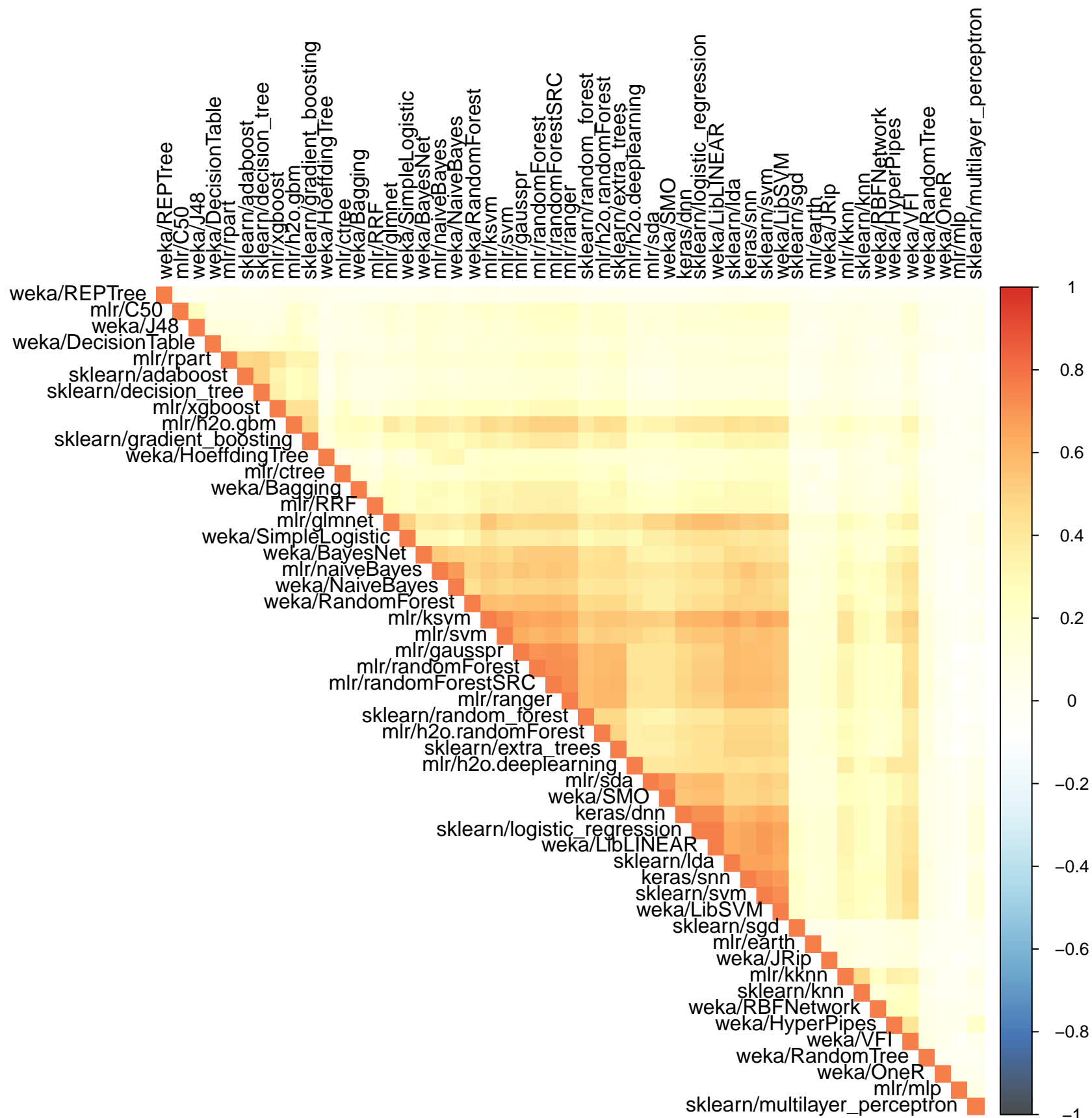

Supplement: S7 Fig — We used each classification algorithm to make probabilistic predictions of relapse in Wilms tumor patients (GSE10320). Based on these predictions, we calculated the Spearman correlation coefficient for each pair of algorithms. These coefficients, averaged across Monte Carlo cross-validation iterations, are illustrated as a correlation plot, clustered based on similarity. (PDF) [file pcbi.1009926.s007.pdf]

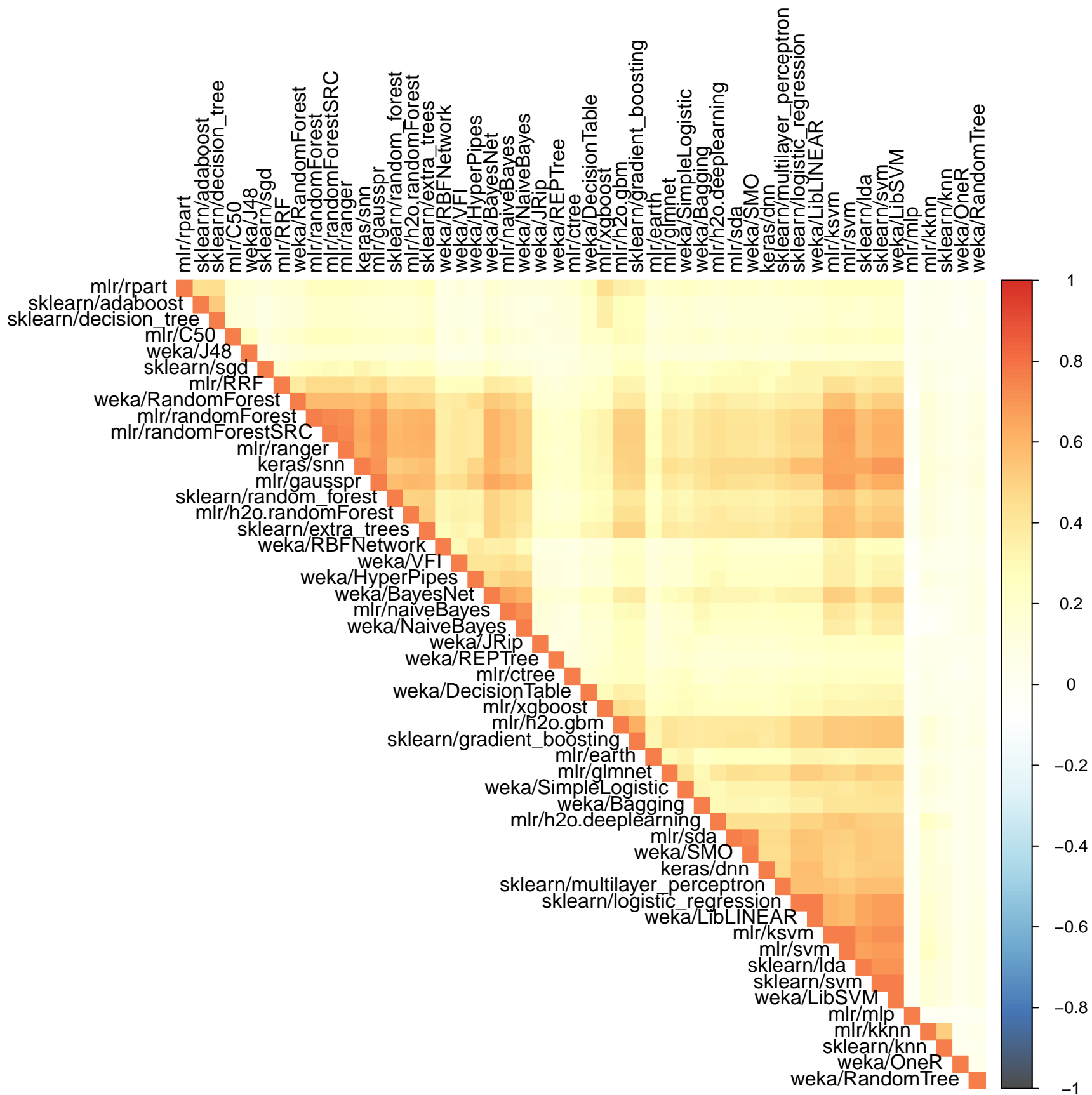

Supplement: S8 Fig — We used each classification algorithm to make probabilistic predictions of early metastasis following radical prostatectomy (GSE46691). Based on these predictions, we calculated the Spearman correlation coefficient for each pair of algorithms. These coefficients, averaged across Monte Carlo cross-validation iterations, are illustrated as a correlation plot, clustered based on similarity. (PDF) [file pcbi.1009926.s008.pdf]

Class category

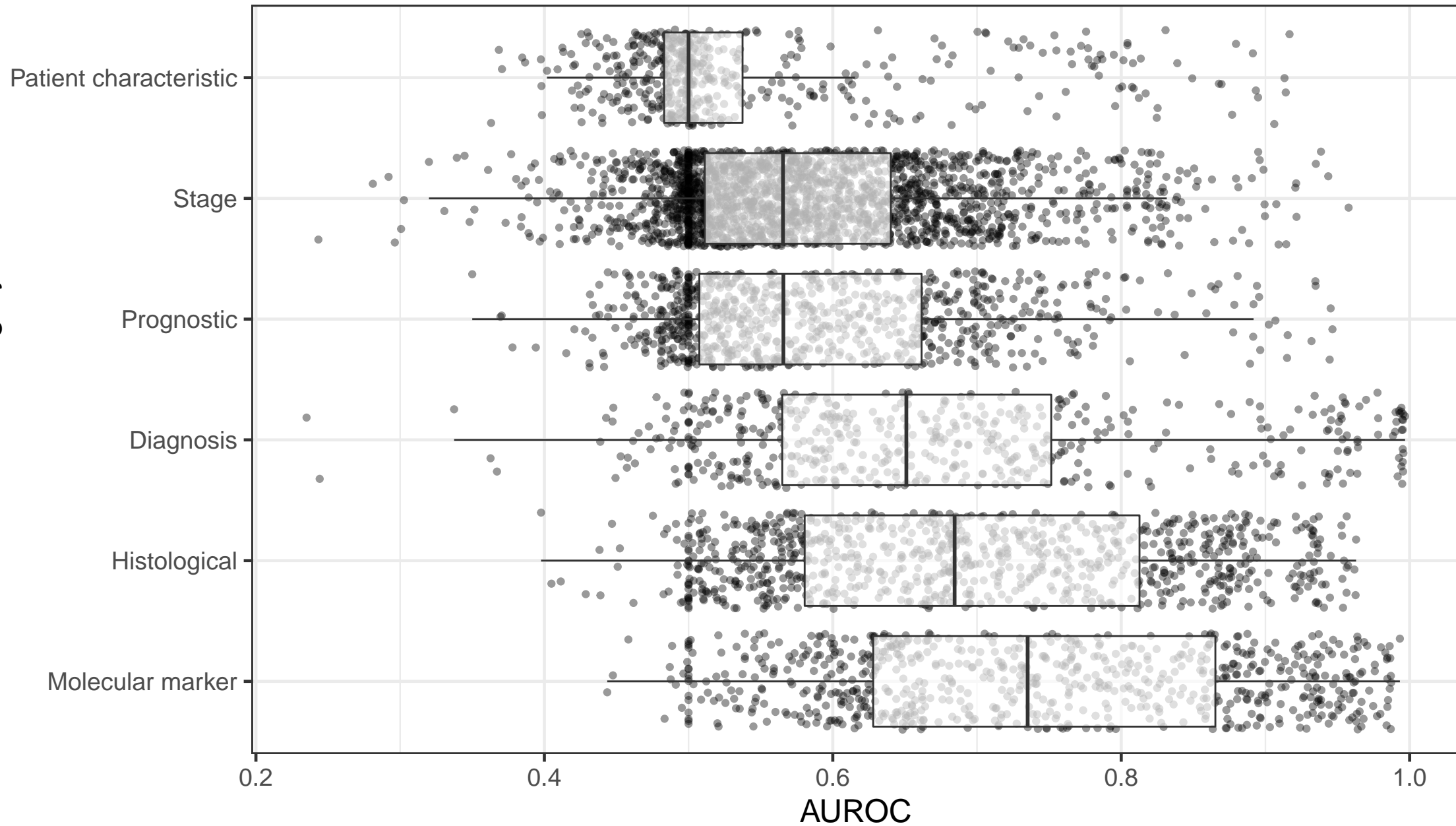

Supplement: S12 Fig — For each class variable across all datasets, we assigned a category representing the type of patient state being predicted. For Analysis 3, we show the predictive performance for each combination of dataset, class variable, and classification algorithm in each class category. We use area under the receiver operating characteristic curve (AUROC) as a metric. The dashed, red line indicates the performance expected by random chance. As with Analysis 1 (S9 Fig), the top-performing category was “Molecular Marker,” which includes class variables associated with mutation status, immunohistochemistry markers of protein expression, presence or absence of chromosomal aberrations, etc. The lowest-performing category was “Patient Characteristic,” which includes variables that indicate whether patients had a family history of cancer, had been diagnosed with multiple tumors, patient performance status, etc. (PDF) [file pcbi.1009926.s012.pdf]

$\rho = 0.13$  ( $p = 0.13$ )

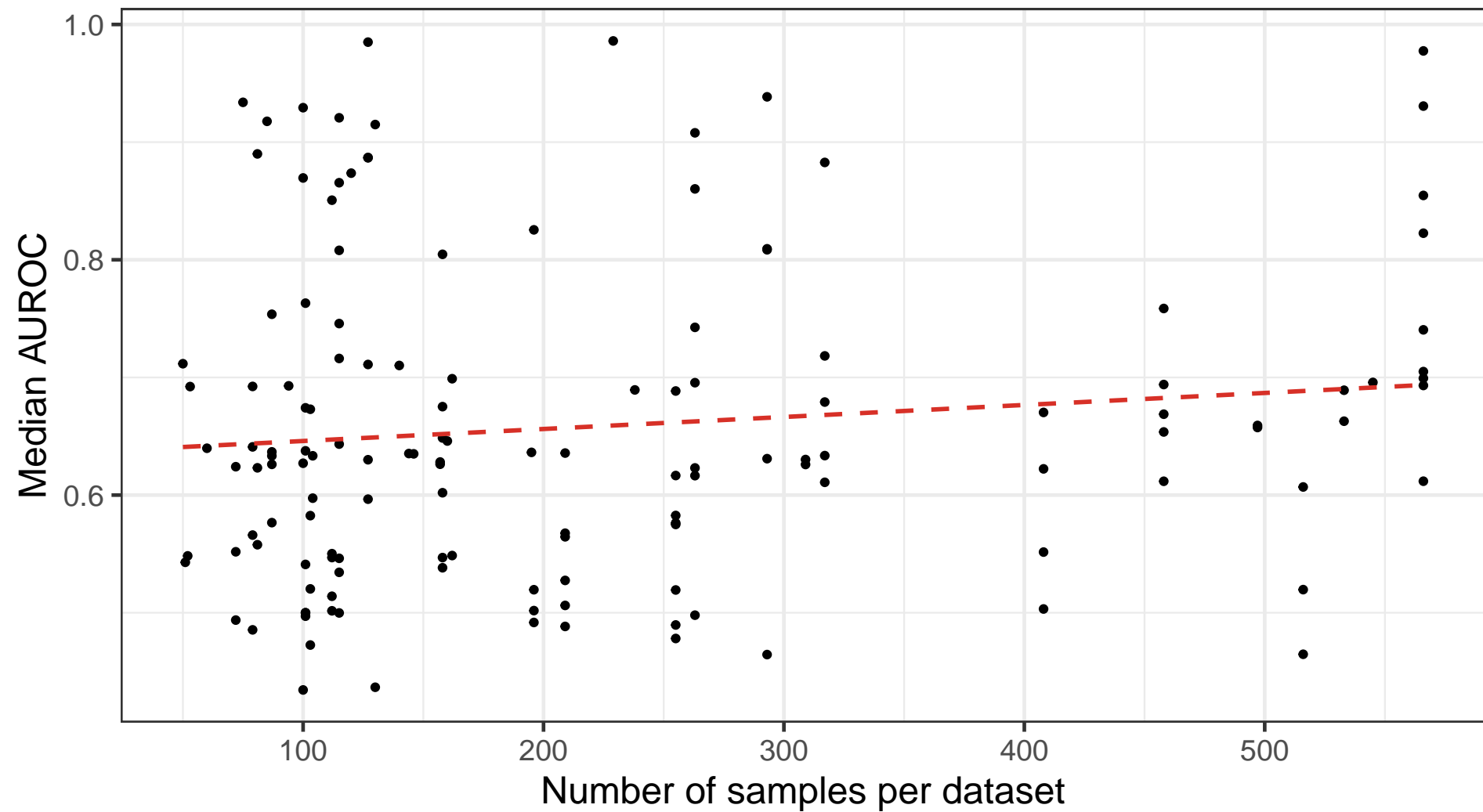

Supplement: S16 Fig — The number of patient samples differed by dataset. This scatterplot shows the relationship between the median area under the receiver operating characteristic curve (AUROC) and the number of samples in each dataset. We did not observe a significant correlation between these variables. (PDF) [file pcbi.1009926.s016.pdf]

$\rho = -0.07$  ( $p = 0.43$ )

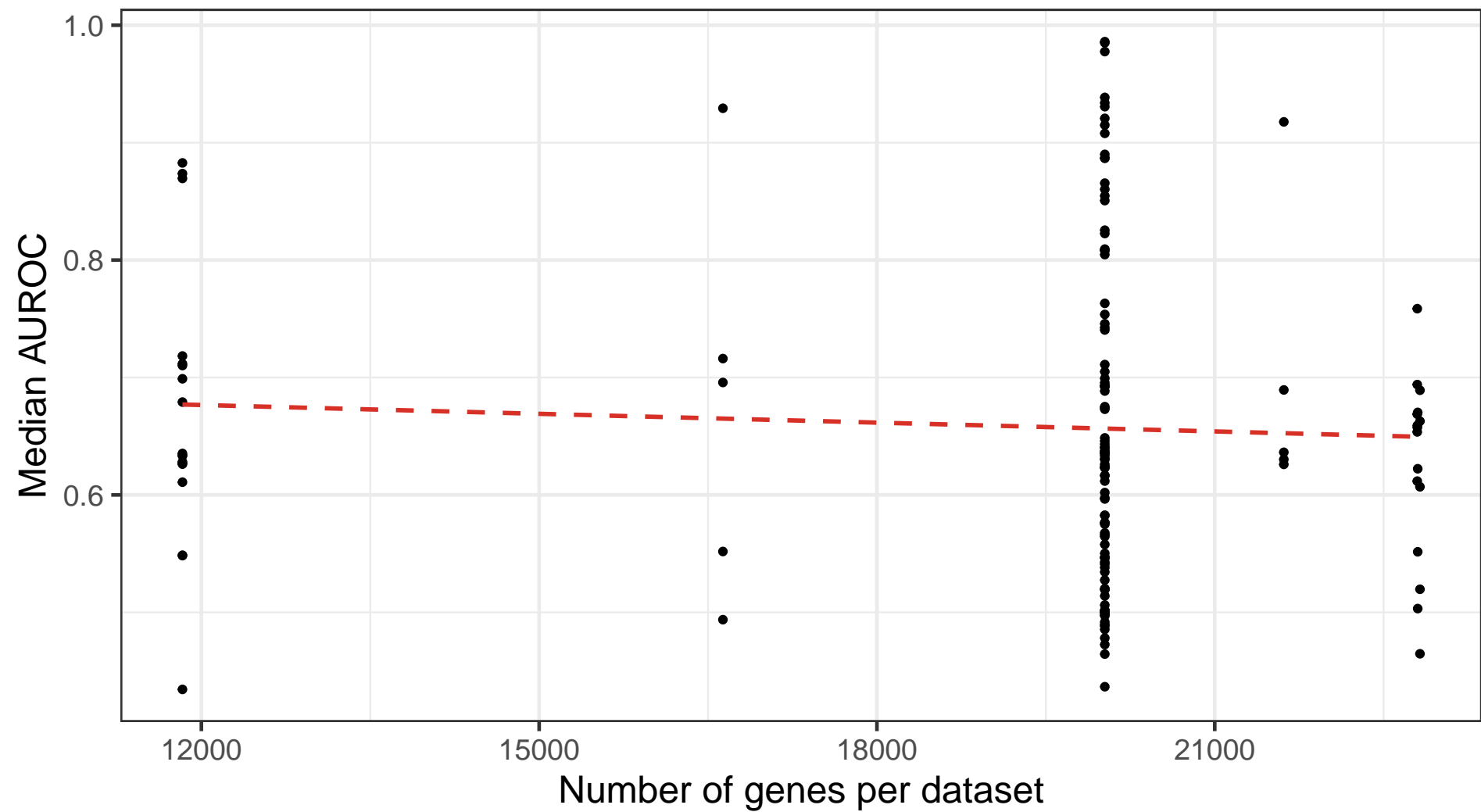

Supplement: S17 Fig — Due to differences in gene-expression profiling platforms, we had data for more genes in some datasets than in others. This scatterplot shows the relationship between the median area under the receiver operating characteristic curve (AUROC) and the number of genes in each dataset. We did not observe a significant correlation between these variables. (PDF) [file pcbi.1009926.s017.pdf]

Dataset / class

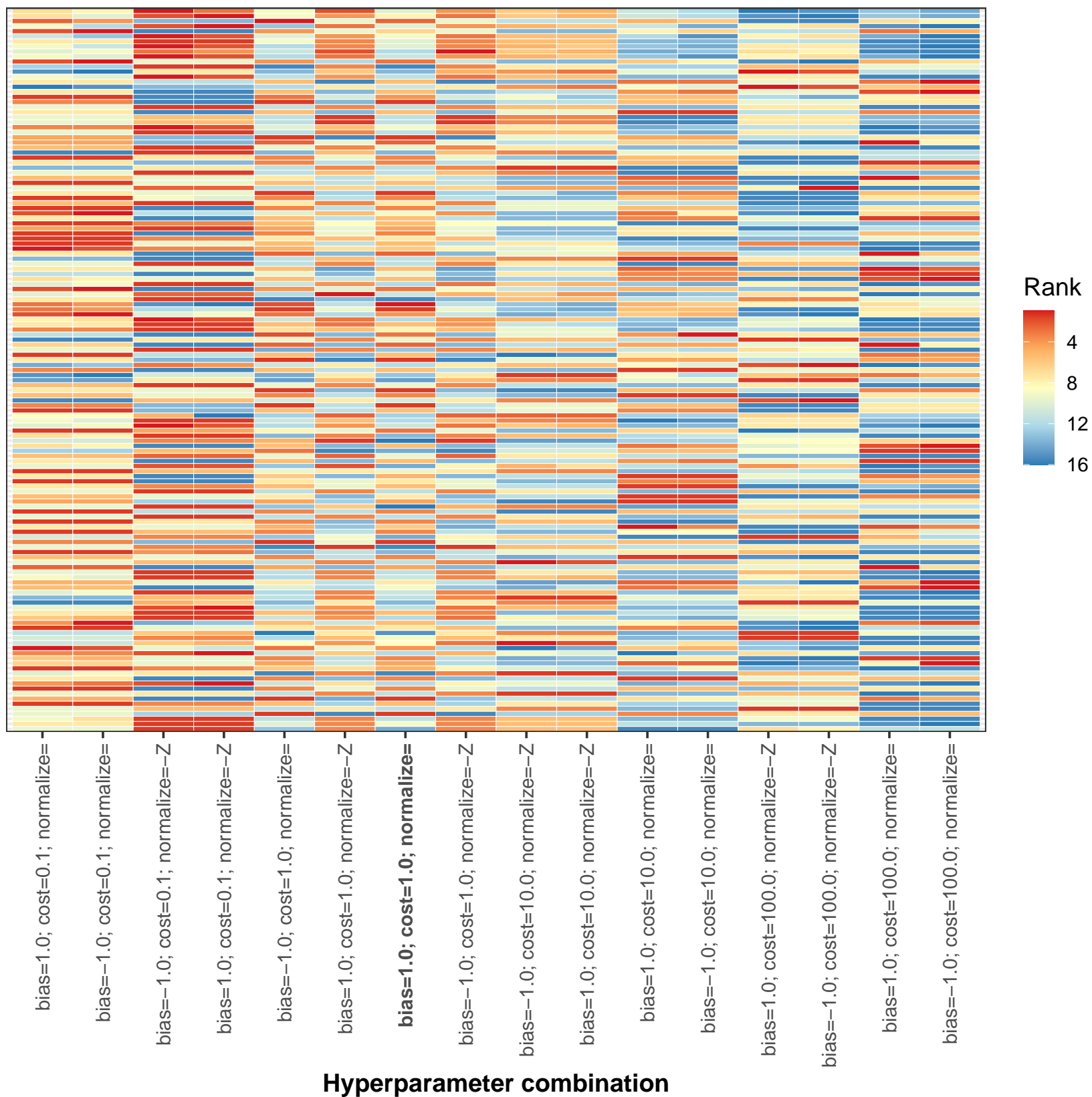

Supplement: S19 Fig — The ShinyLearner software supports 16 hyperparameter combinations for the weka/LIBLINEAR classification algorithm. In Analysis 4, we used nested cross validation for hyperparameter optimization. For each combination of dataset and class variable, we averaged the area under the receiver operating characteristic curve (AUROC) across all (outer) Monte Carlo cross-validation iterations and then ranked the averages for each hyperparameter combination. Some combinations consistently outperformed other combinations, and the default combination performed suboptimally. Using relatively small cost values appeared to improve the performance more than any other option. This hyperparameter controls the regularization strength. (PDF) [file pcbi.1009926.s019.pdf]

Dataset / class

alpha=0

alpha=0.5

alpha=1

Hyperparameter combination

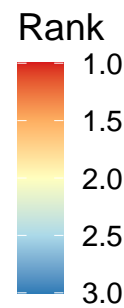

Supplement: S20 Fig — The ShinyLearner software supports 3 hyperparameter combinations for the mlr/glmnet classification algorithm. In Analysis 4, we used nested cross validation for hyperparameter optimization. For each combination of dataset and class variable, we averaged the area under the receiver operating characteristic curve (AUROC) across all (outer) Monte Carlo cross-validation iterations and then ranked the averages for each hyperparameter combination. Using an alpha value of 0.5 or 0 resulted in better performance than a value of 1. (PDF) [file pcbi.1009926.s020.pdf]

Dataset / class

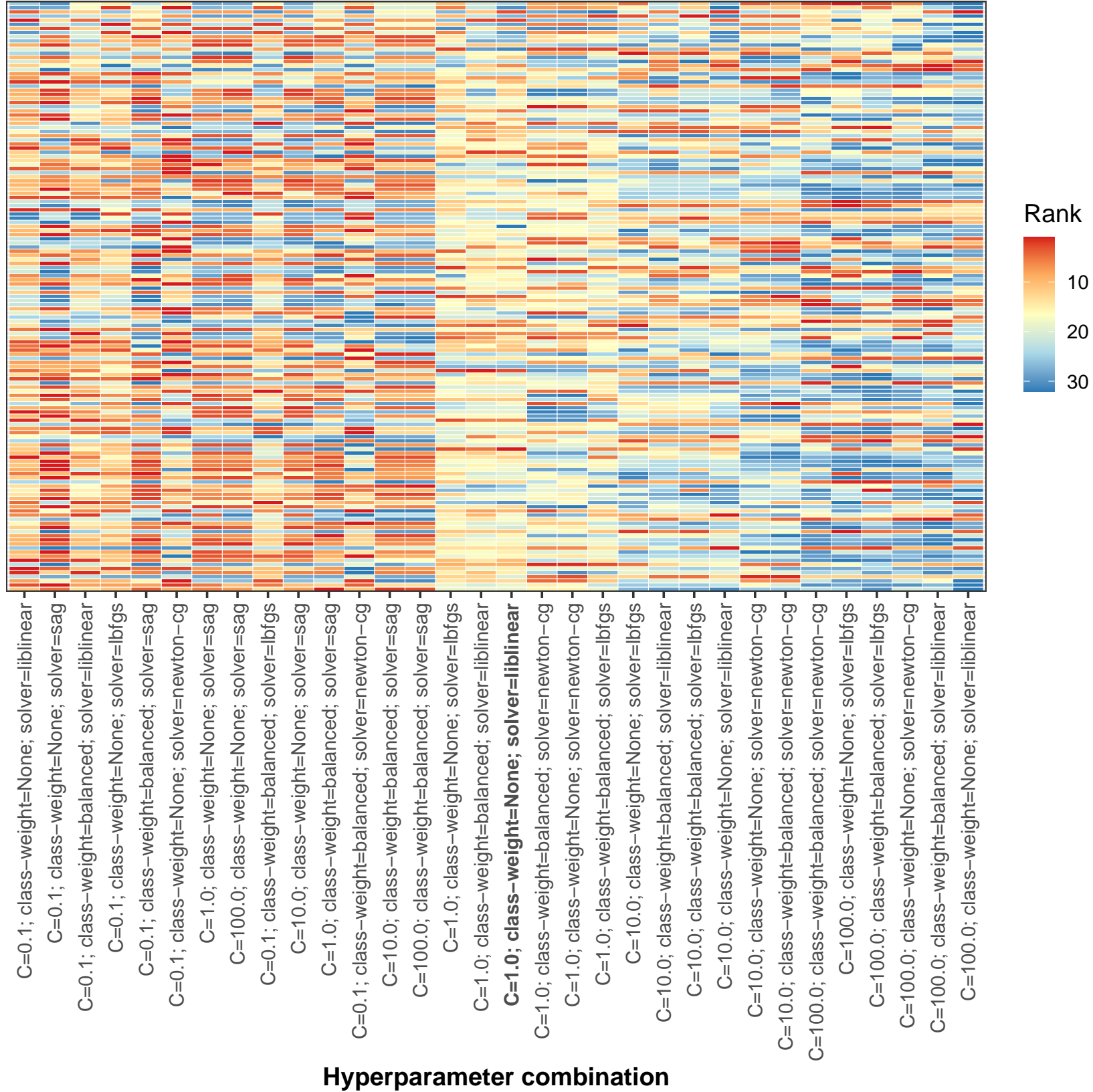

Supplement: S21 Fig — The ShinyLearner software supports 32 hyperparameter combinations for the sklearn/logistic_regression classification algorithm. In Analysis 4, we used nested cross validation for hyperparameter optimization. For each combination of dataset and class variable, we averaged the area under the receiver operating characteristic curve (AUROC) across all (outer) Monte Carlo cross-validation iterations and then ranked the averages for each hyperparameter combination. Some combinations consistently outperformed other combinations, and the default combination performed suboptimally. Using relatively small cost values appeared to improve the performance more than any other option. This hyperparameter controls the regularization strength. (PDF) [file pcbi.1009926.s021.pdf]

## Hyperparameter combination

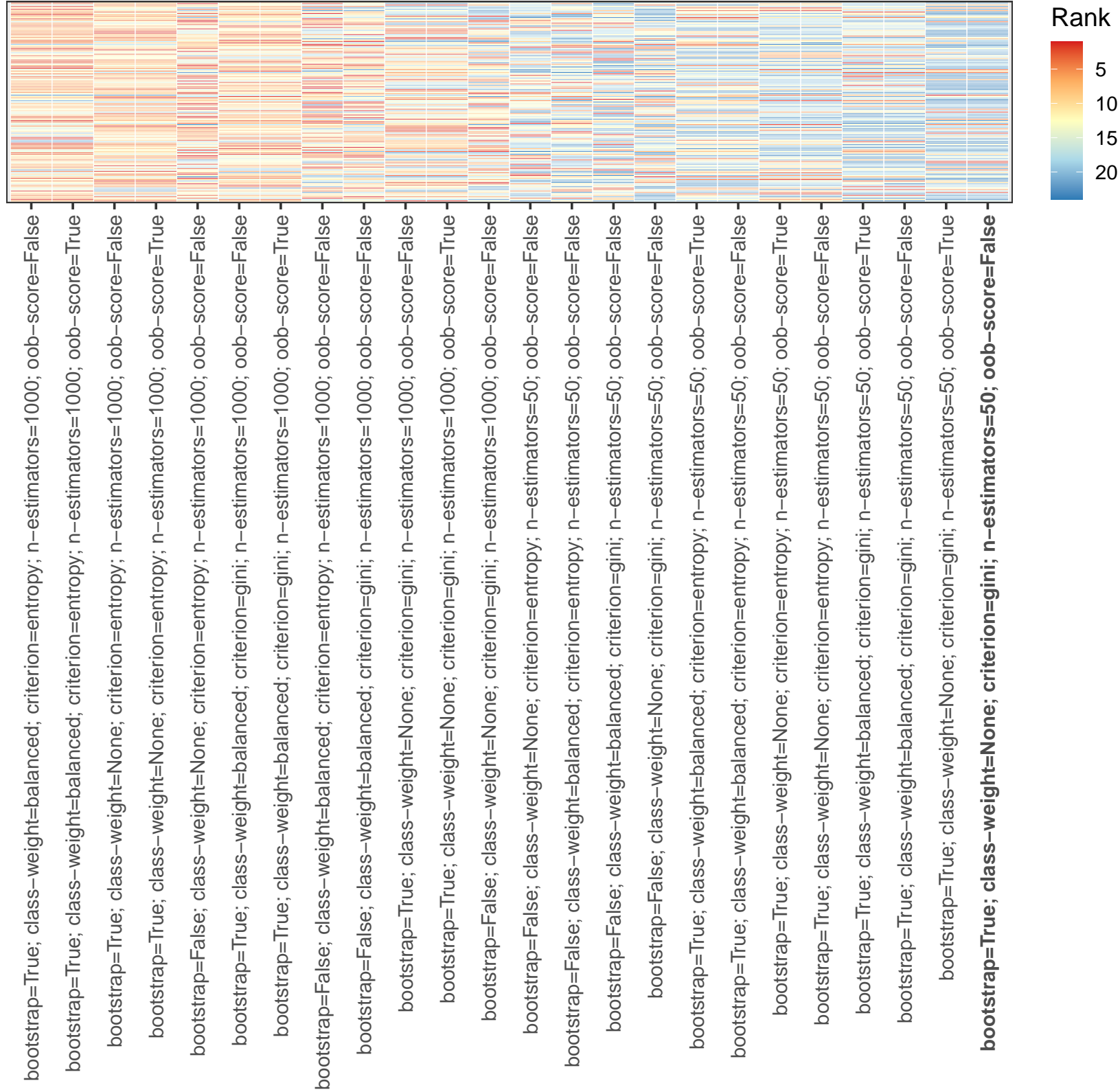

Supplement: S22 Fig — The ShinyLearner software supports 24 hyperparameter combinations for the sklearn/extra_trees classification algorithm. In Analysis 4, we used nested cross validation for hyperparameter optimization. For each combination of dataset and class variable, we averaged the area under the receiver operating characteristic curve (AUROC) across all (outer) Monte Carlo cross-validation iterations and then ranked the averages for each hyperparameter combination. Some combinations consistently outperformed other combinations, and the default combination performed suboptimally. Using a larger number (n = 1000) of estimators (trees) appeared to improve the performance more than any other option. (PDF) [file pcbi.1009926.s022.pdf]

Spearman's rho = 0.73

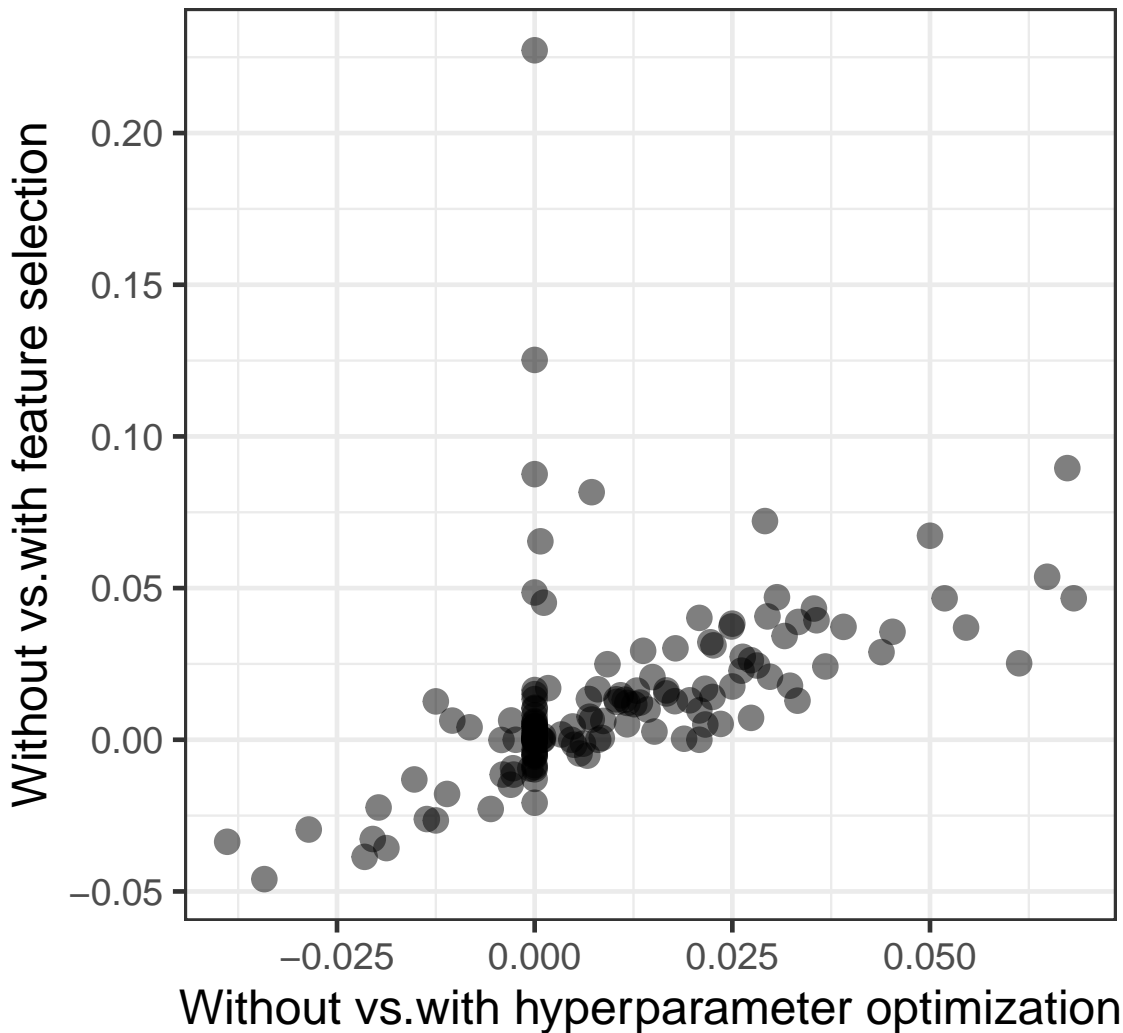

Supplement: S23 Fig — We used as a baseline the predictive performance that we attained using default hyperparameters for the classification algorithms (Analysis 3). We quantified predictive performance using the area under the receiver operating characteristic curve (AUROC). This graph shows the increase or decrease in performance when selecting hyperparameters or selecting features relative to the baseline. Each point represents a particular combination of dataset and class variable. Generally, the dataset/class combinations that benefitted from hyperparameter optimization also benefitted from feature selection. However, some dataset/class combinations that did not benefit from hyperparameter optimization did benefit from feature selection. (PDF) [file pcbi.1009926.s023.pdf]

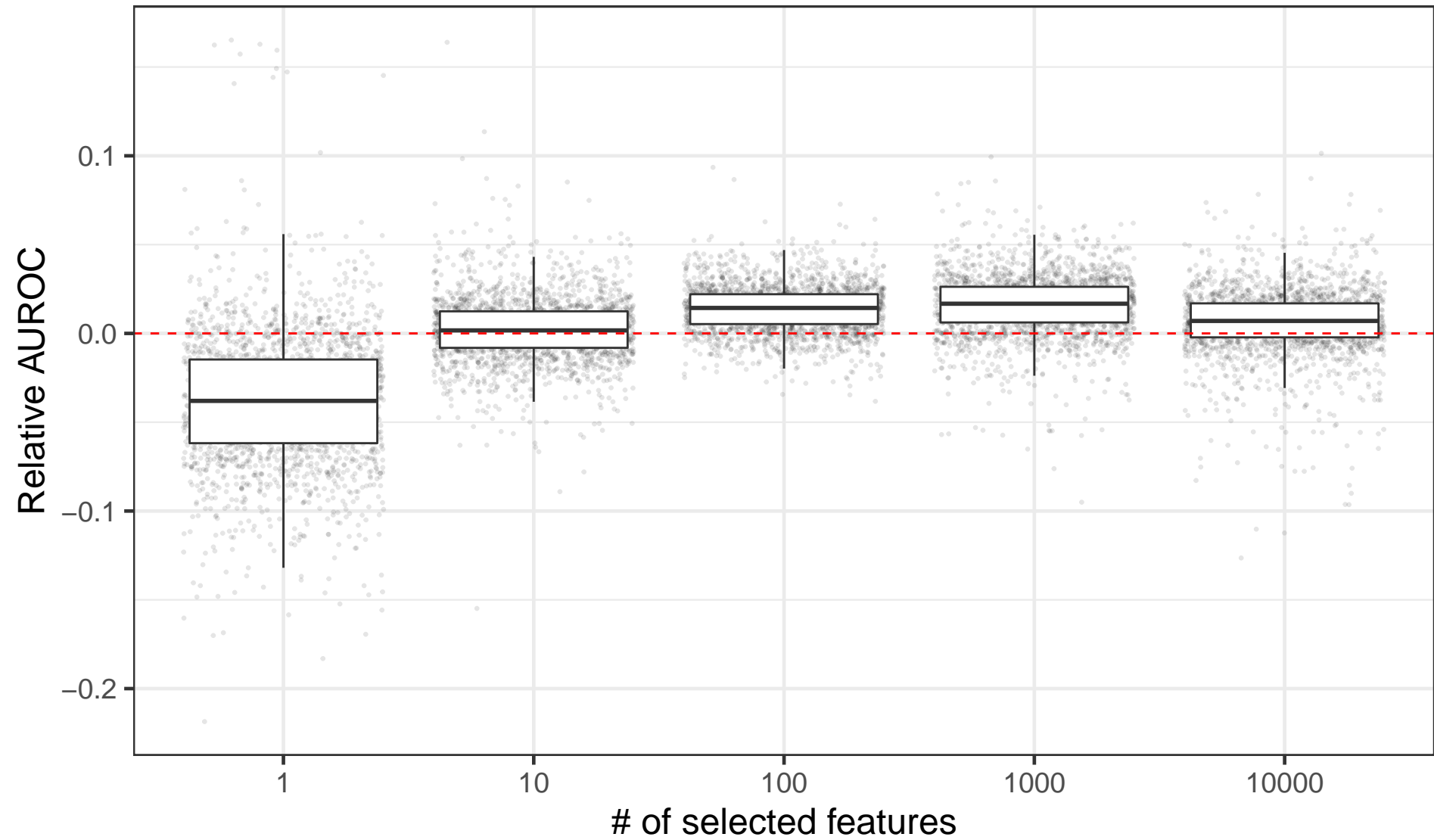

Supplement: S25 Fig — Relative area under the receiver operating character curve (AUROC) values were calculated by comparing against the mean for each combination of classification algorithm and feature-selection algorithm. (PDF) [file pcbi.1009926.s025.pdf]

Feature-selection algorithm

Univariate  
Multivariate

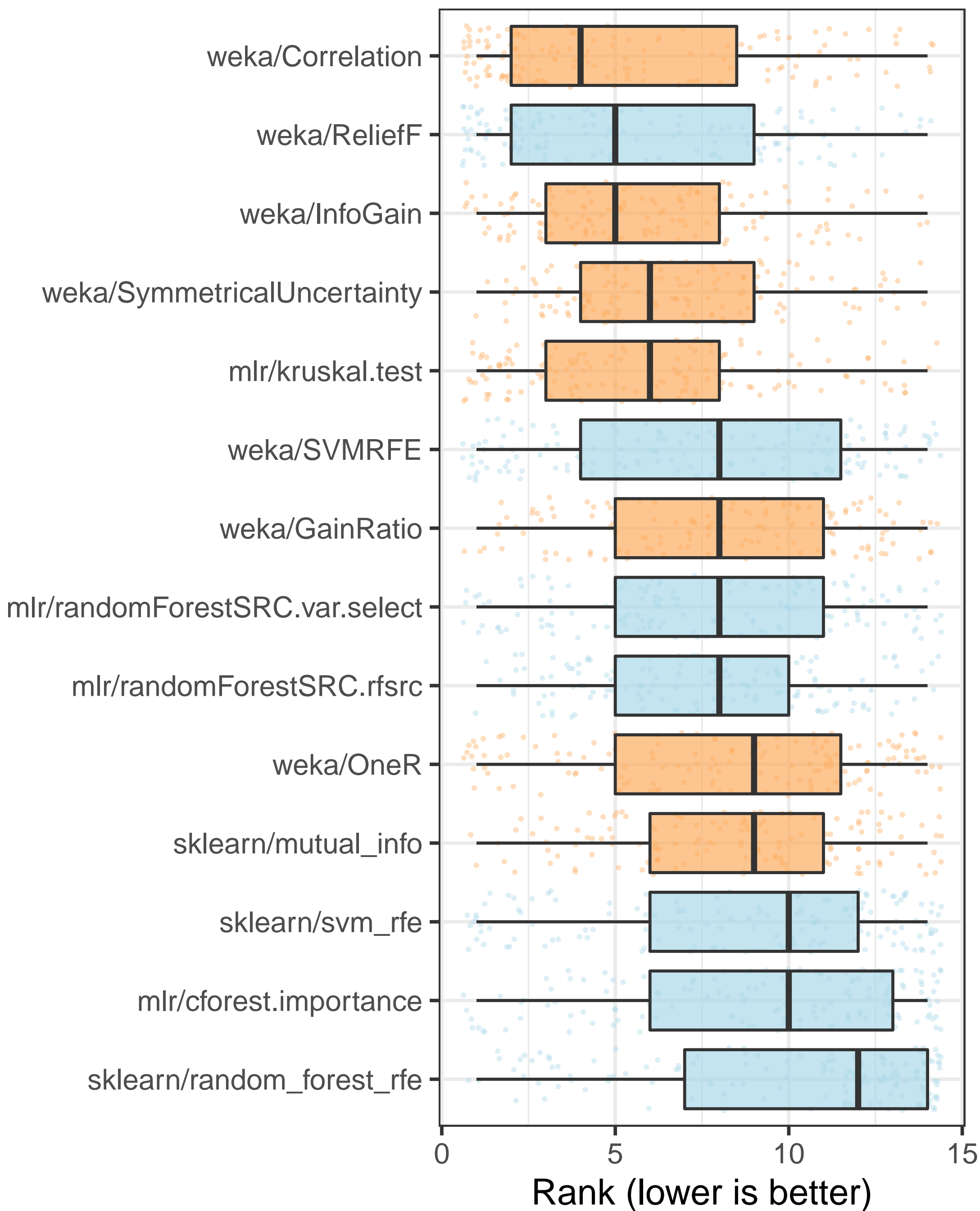

Supplement: S26 Fig — For Analysis 5, we used nested cross validation to estimate which features would be most informative for each algorithm in each training set. For each combination of dataset, class variable, and classification algorithm, we ranked the performance of the feature-selection algorithms based on area under the receiver operating characteristic curve (AUROC) and averaged the rankings across 5 iterations of Monte Carlo cross-validation. Each data point that overlays the box plots represents a particular dataset/class combination. Relatively low average ranks are considered optimial. The weka/Correlation feature-selection algorithm performed best overall. (PDF) [file pcbi.1009926.s026.pdf]

Feature Selection Algorithm

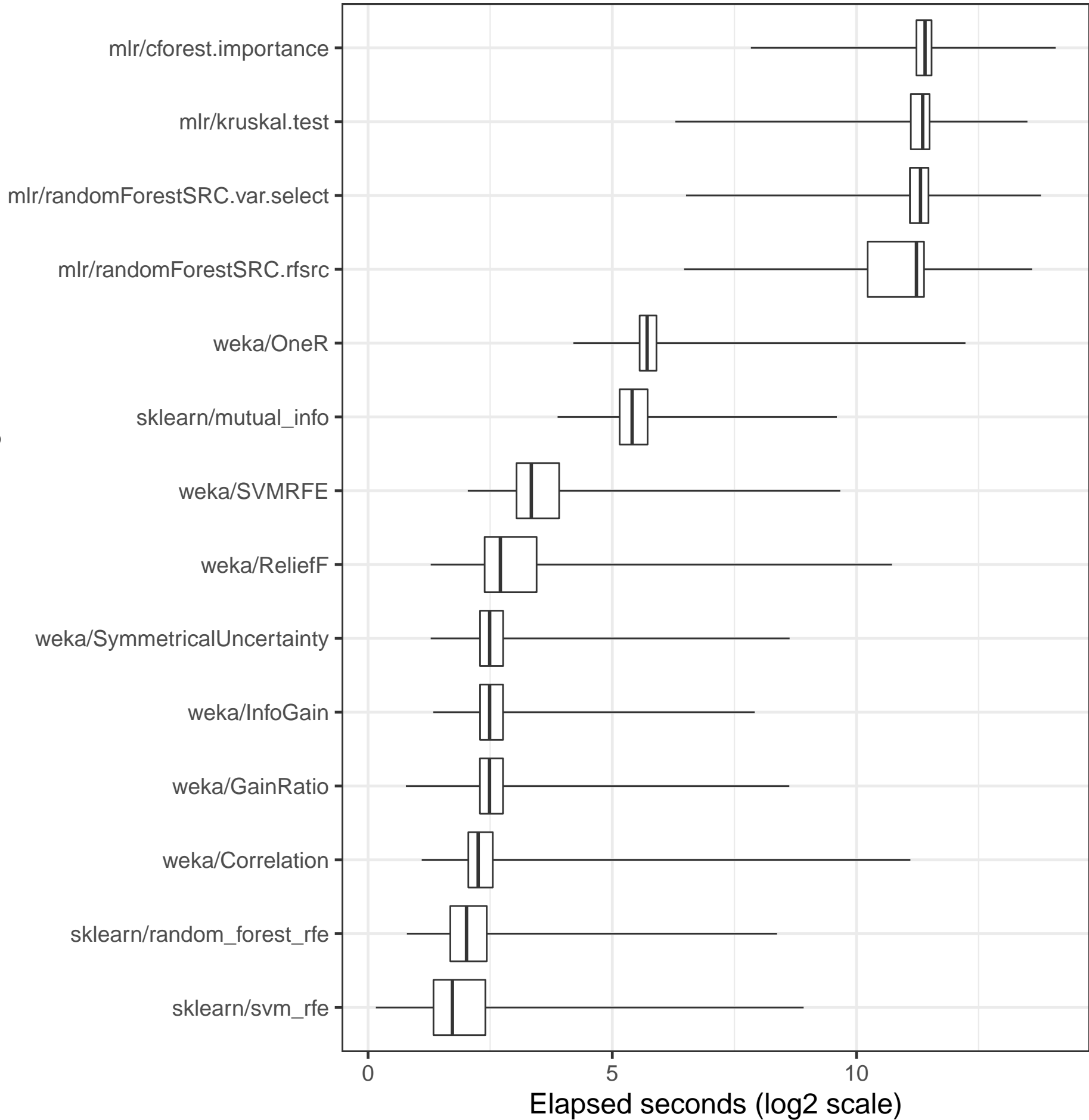

Supplement: S27 Fig — In Analysis 5, we used nested cross validation to estimate which features were most informative for each training set. We calculated the time (in seconds) required by each feature-selection algorithm to rank the features. Then we averaged these times across all combinations of dataset, class variable, classification algorithm, and (outer) Monte Carlo cross-validation iteration. Some feature-selection algorithms were much more computationally intensive than others. (PDF) [file pcbi.1009926.s027.pdf]

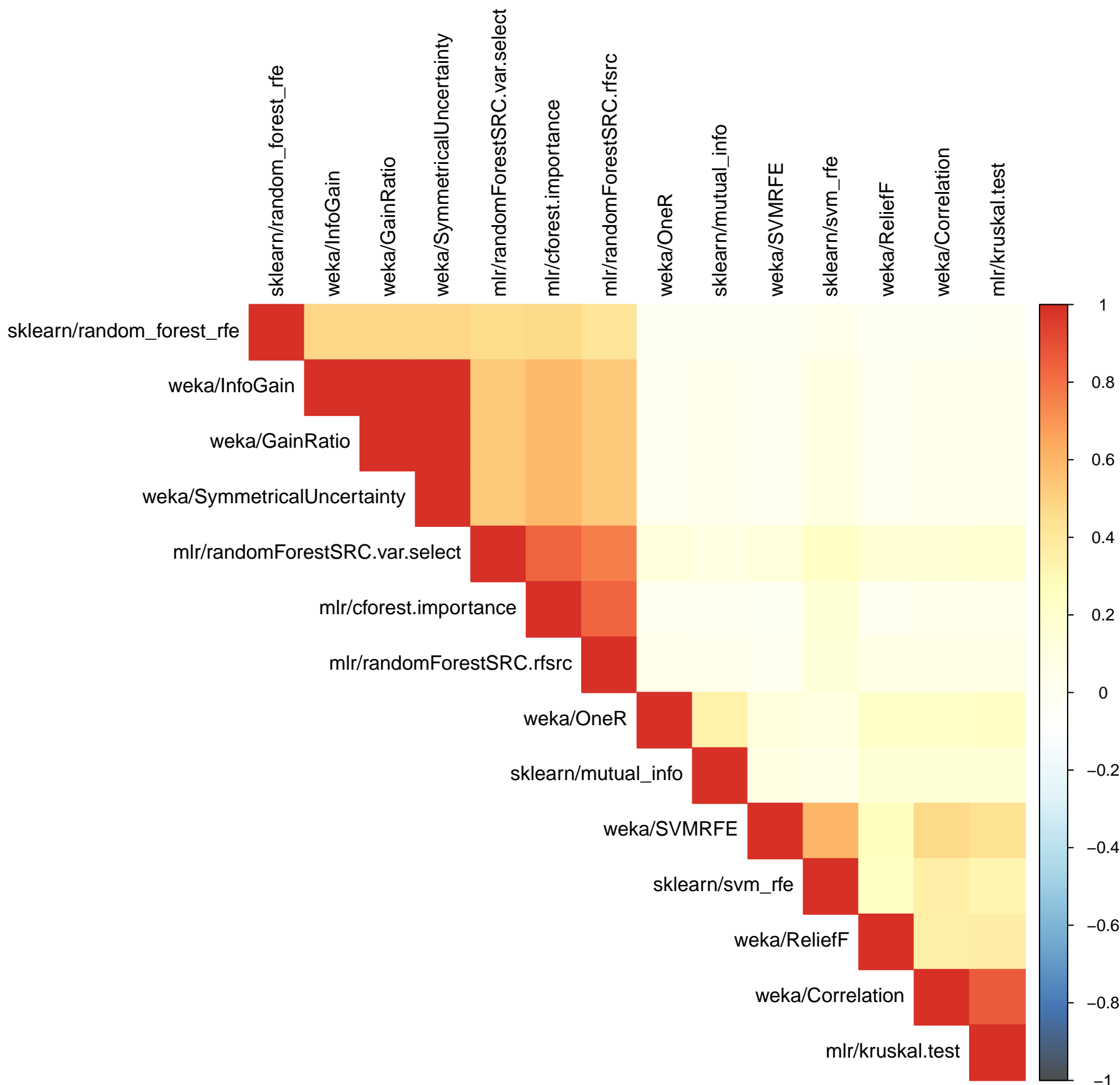

Supplement: S28 Fig — We used each feature-selection algorithm to rank the genes based on their informativeness for discriminating between relapse and non-relapse outcomes in Wilms tumor patients (GSE10320). After averaging the ranks across cross-validation iterations, we calculated the Spearman correlation coefficient for the feature ranks produced by each pair of algorithms. These coefficients are illustrated as a correlation plot. (PDF) [file pcbi.1009926.s028.pdf]

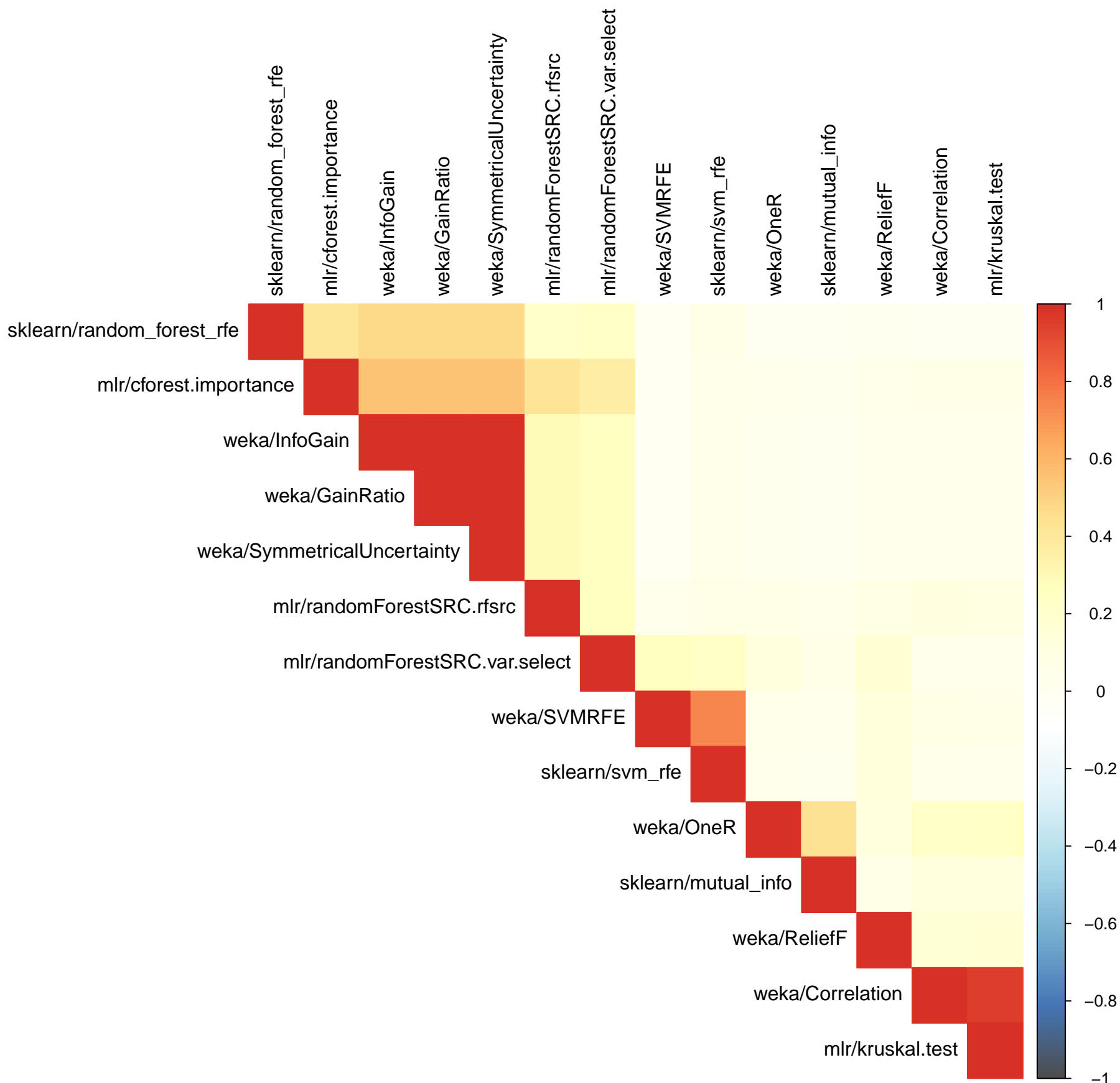

Supplement: S29 Fig — We used each feature-selection algorithm to rank the genes based on their informativeness for predicting early metastasis following radical prostatectomy (GSE46691). After averaging the ranks across cross-validation iterations, we calculated the Spearman correlation coefficient for the feature ranks produced by each pair of algorithms. These coefficients are illustrated as a correlation plot. (PDF) [file pcbi.1009926.s029.pdf]

Feature Selection Algorithm

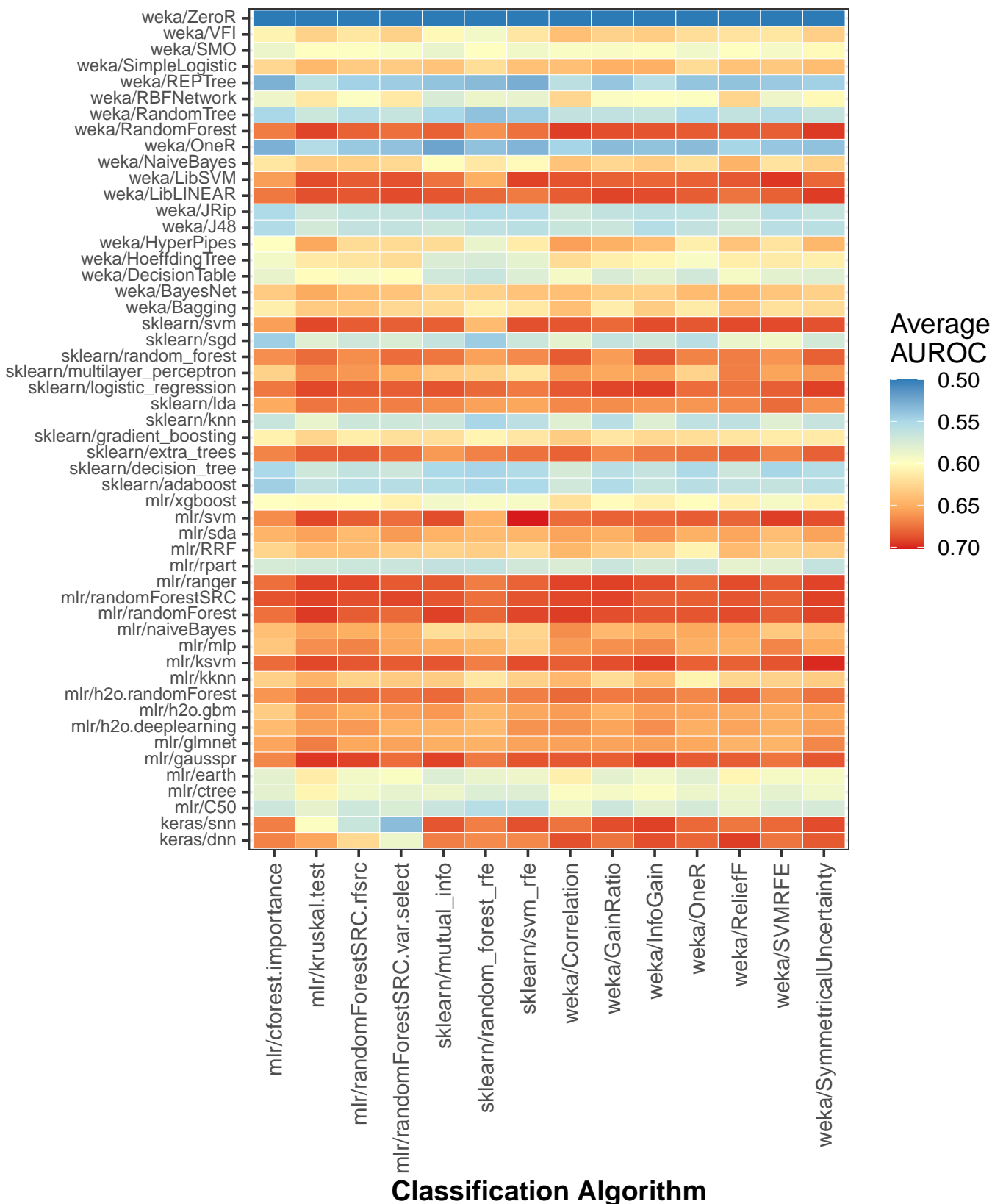

Supplement: S30 Fig — For each combination of dataset and class variable, we averaged the area under the receiver operating characteristic curve (AUROC) across all Monte Carlo cross-validation iterations. Then for each combination of feature-selection algorithm and classification algorithm, we calculated the median AUROC across all datasets and class variables. (PDF) [file pcbi.1009926.s030.pdf]

# GSE10320

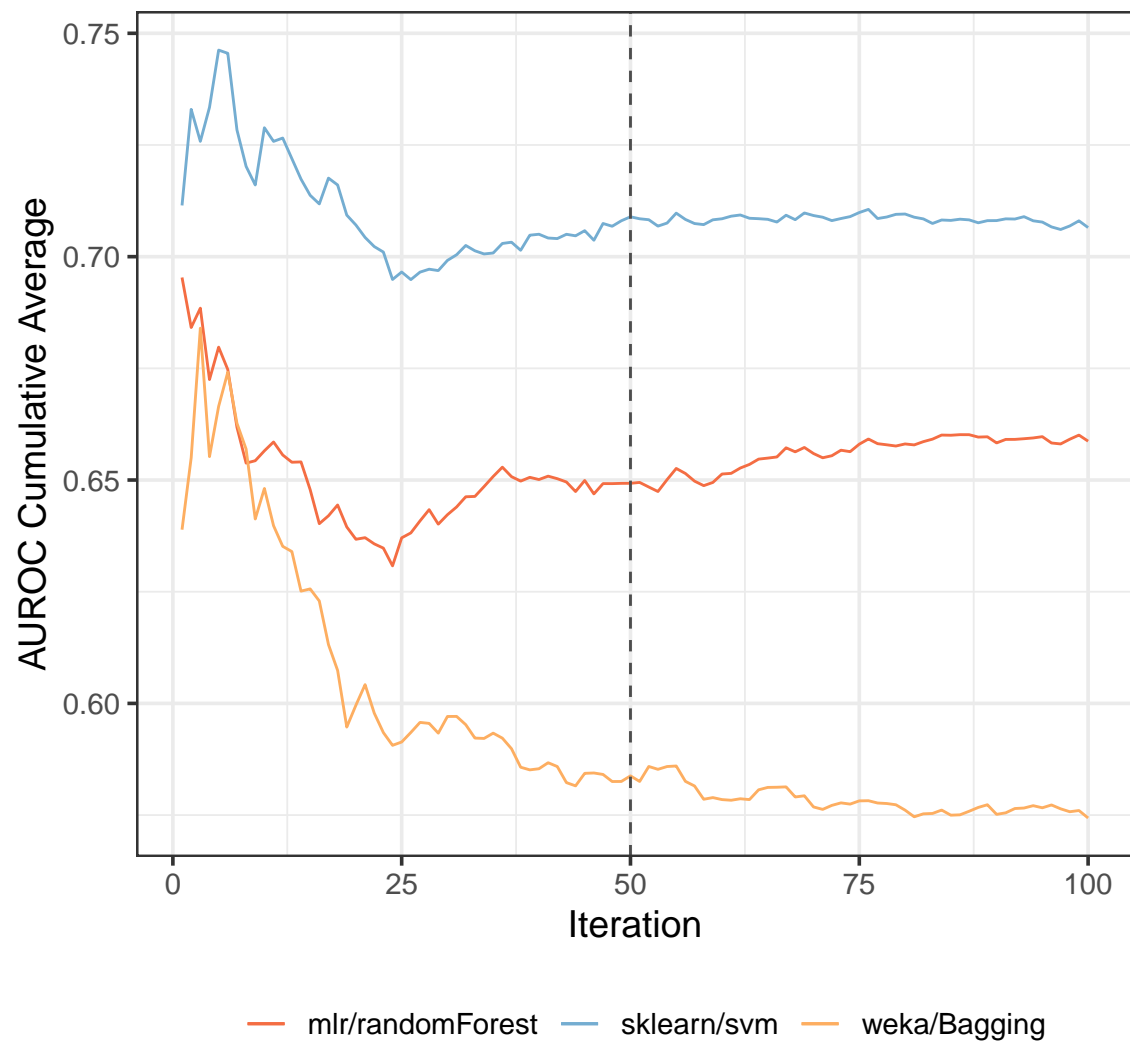

Supplement: S32 Fig — When using gene-expression predictors (Analysis 1), we estimated the number of Monte Carlo cross-validation iterations that would be sufficient to characterize algorithm performance. For three classification algorithms, we executed 100 cross-validation iterations on dataset GSE10320 (predicting relapse vs. non-relapse for Wilms tumor patients). As the number of iterations increased, we calculated the cumulative average of the area under the receiver operating characteristic curve (AUROC) for each algorithm. After performing at most 40 iterations, the cumulative averages did not change more than 0.01 over sequences of 10 iterations. (PDF) [file pcbi.1009926.s032.pdf]

# GSE46691

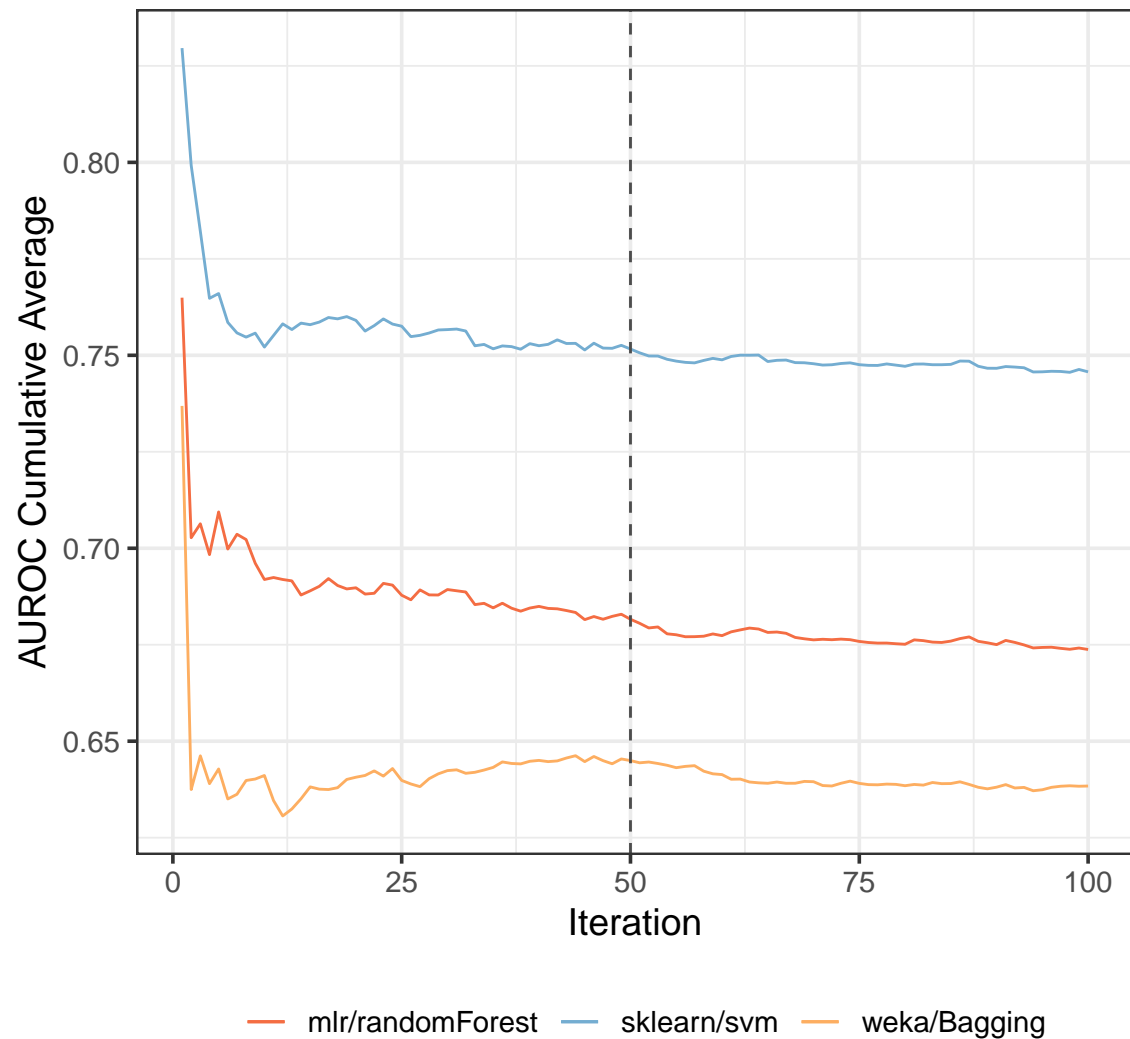

Supplement: S33 Fig — When using gene-expression predictors (Analysis 1), we estimated the number of Monte Carlo cross-validation iterations that would be sufficient to characterize algorithm performance. For three classification algorithms, we executed 100 cross-validation iterations on dataset GSE46691 (predicting early metastasis following radical prostatectomy). As the number of iterations increased, we calculated the cumulative average of the area under the receiver operating characteristic curve (AUROC) for each algorithm. After performing at most 22 iterations, the cumulative averages did not change more than 0.01 over sequences of 10 iterations. (PDF) [file pcbi.1009926.s033.pdf]
