# Supplementary material for: The ability to classify patients based on gene-expression data varies by algorithm and performance metric
Source: PLoS Comput Biol. 2022 Mar 11;18(3):e1009926. doi: 10.1371/journal.pcbi.1009926 (PMC8942277; doi:10.1371/journal.pcbi.1009926)
Supplement: S10 Fig — We predicted patient states using clinical predictors only (Analysis 2). For each combination of dataset, class variable, and classification algorithm, we calculated the arithmetic mean of area under the receiver operating characteristic curve (AUROC) values across 50 iterations of Monte Carlo cross-validation. Next, we sorted the algorithms based on the average rank across all dataset/class combinations. Each data point that overlays the box plots represents a particular dataset/class combination (some datasets did not have clinical predictors). The top-performing algorithms (relatively low ranks) were similar overall to Analysis 1; however, some differences were large. For example, weka/NaiveBayes performed best overall in Analysis 2 but was ranked 28th in Analysis 1. (PDF) [file pcbi.1009926.s010.pdf]

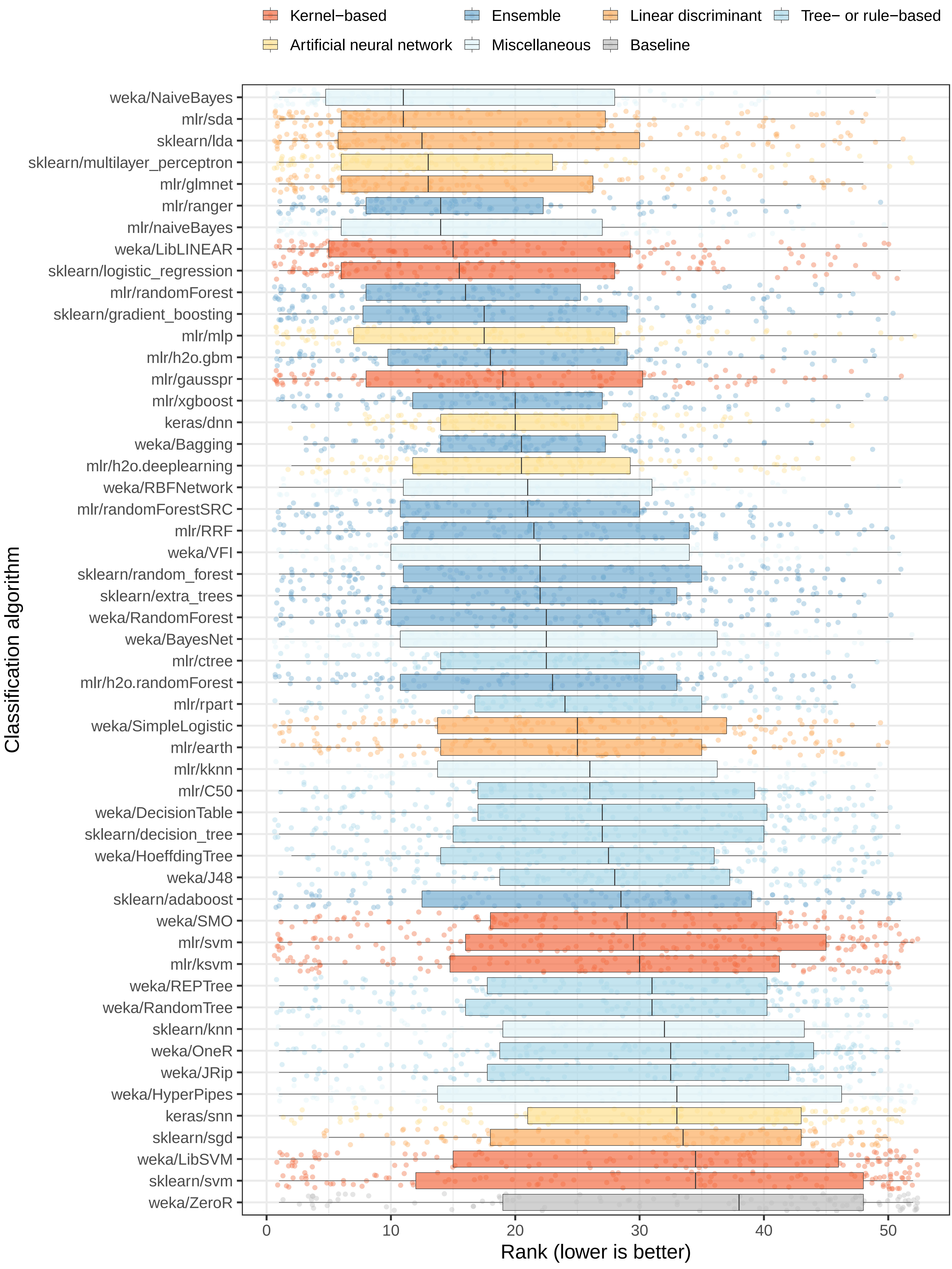

Kernel−based
Ensemble
Linear discriminant
Tree− or rule−based
Artificial neural network
Miscellaneous
Baseline
weka/NaiveBayes
mlr/sda
sklearn/lda
sklearn/multilayer_perceptron
mlr/glmnet
mlr/ranger
mlr/naiveBayes
weka/LibLINEAR
sklearn/logistic_regression
mlr/randomForest
sklearn/gradient_boosting
mlr/mlp
mlr/h2o.gbm
mlr/gausspr
mlr/xgboost
keras/dnn
weka/Bagging
mlr/h2o.deeplearning
weka/RBFNetwork
mlr/randomForestSRC
mlr/RRF
weka/VFI
sklearn/random_forest
sklearn/extra_trees
weka/RandomForest
weka/BayesNet
mlr/ctree
mlr/h2o.randomForest
mlr/rpart
weka/SimpleLogistic
mlr/earth
mlr/kknn
mlr/C50
weka/DecisionTable
sklearn/decision_tree
weka/HoeffdingTree
weka/J48
sklearn/adaboost
weka/SMO
mlr/svm
mlr/ksvm
weka/REPTree
weka/RandomTree
sklearn/knn
weka/OneR
weka/JRip
weka/HyperPipes
keras/snn
sklearn/sgd
weka/LibSVM
sklearn/svm
weka/ZeroR
Classification algorithm
0
10
20
30
40
50
Rank (lower is better)
