# Supplementary material for: The ability to classify patients based on gene-expression data varies by algorithm and performance metric
Source: PLoS Comput Biol. 2022 Mar 11;18(3):e1009926. doi: 10.1371/journal.pcbi.1009926 (PMC8942277; doi:10.1371/journal.pcbi.1009926)
Supplement: S11 Fig — For each class variable across all datasets, we assigned a category representing the type of patient state being predicted. For Analysis 2, we show the predictive performance for each combination of dataset, class variable, and classification algorithm in each class category. We use area under the receiver operating characteristic curve (AUROC) as the metric. The dashed, red line indicates the performance expected by random chance. The top-performing category was “Diagnosis,” which includes class variables associated with a particular disease or subtype. The lowest-performing category was “Patient Characteristic,” which includes variables that indicate whether patients had a family history of cancer, had been diagnosed with multiple tumors, patient performance status, etc. (PDF) [file pcbi.1009926.s011.pdf]

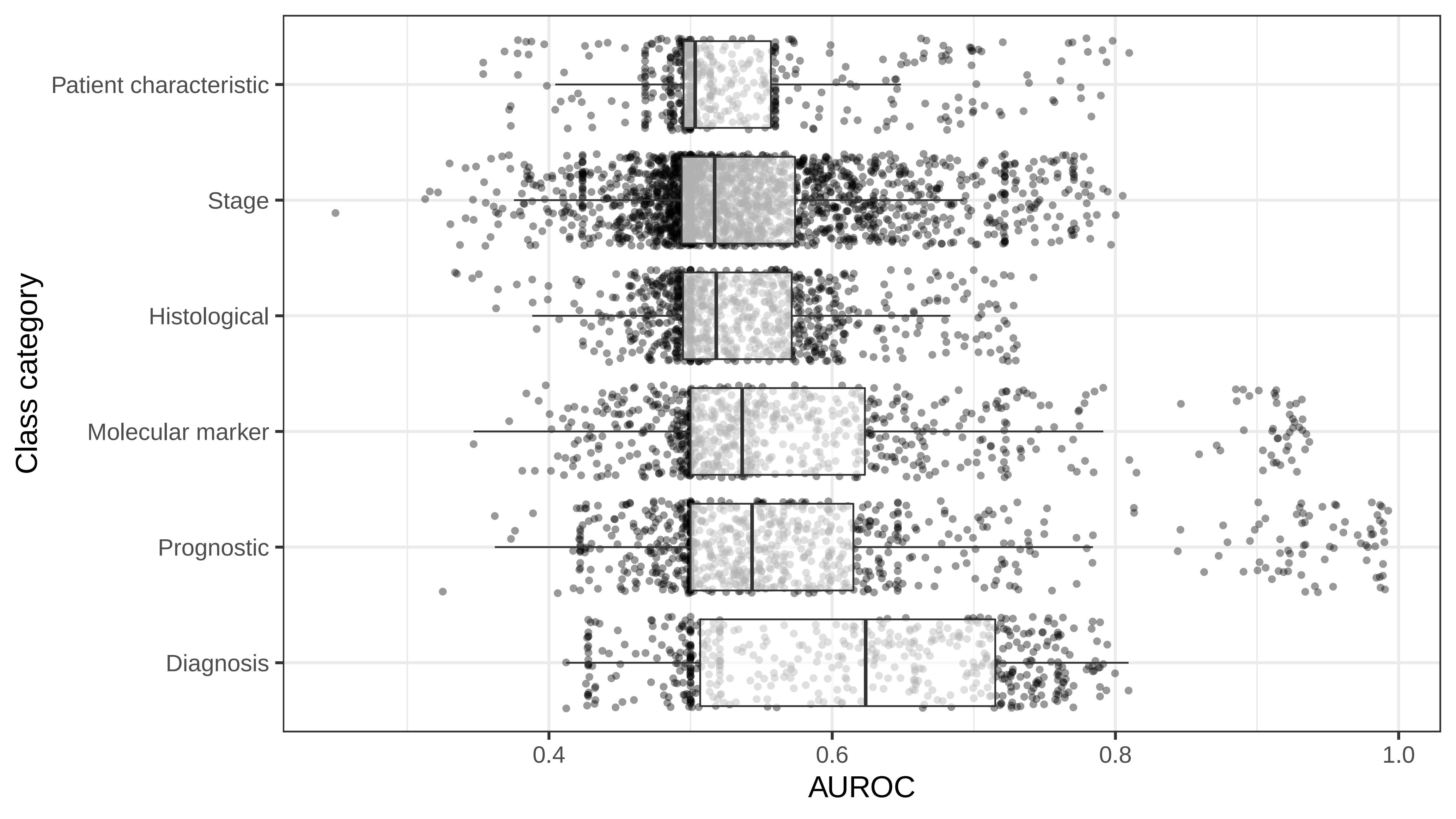
Patient characteristic
Stage
Histological
Molecular marker
Prognostic
Diagnosis
Class category
0.4
0.6
0.8
1.0
AUROC
