# Supplementary material for: The ability to classify patients based on gene-expression data varies by algorithm and performance metric
Source: PLoS Comput Biol. 2022 Mar 11;18(3):e1009926. doi: 10.1371/journal.pcbi.1009926 (PMC8942277; doi:10.1371/journal.pcbi.1009926)
Supplement: S13 Fig — We predicted patient states using gene-expression and clinical predictors (Analysis 3). For each combination of dataset, class variable, and classification algorithm, we calculated the arithmetic mean of area under the receiver operating characteristic curve (AUROC) values across 50 iterations of Monte Carlo cross-validation. Next, we sorted the algorithms based on the average rank across all dataset/class combinations. Each data point that overlays the box plots represents a particular dataset/class combination. (PDF) [file pcbi.1009926.s013.pdf]

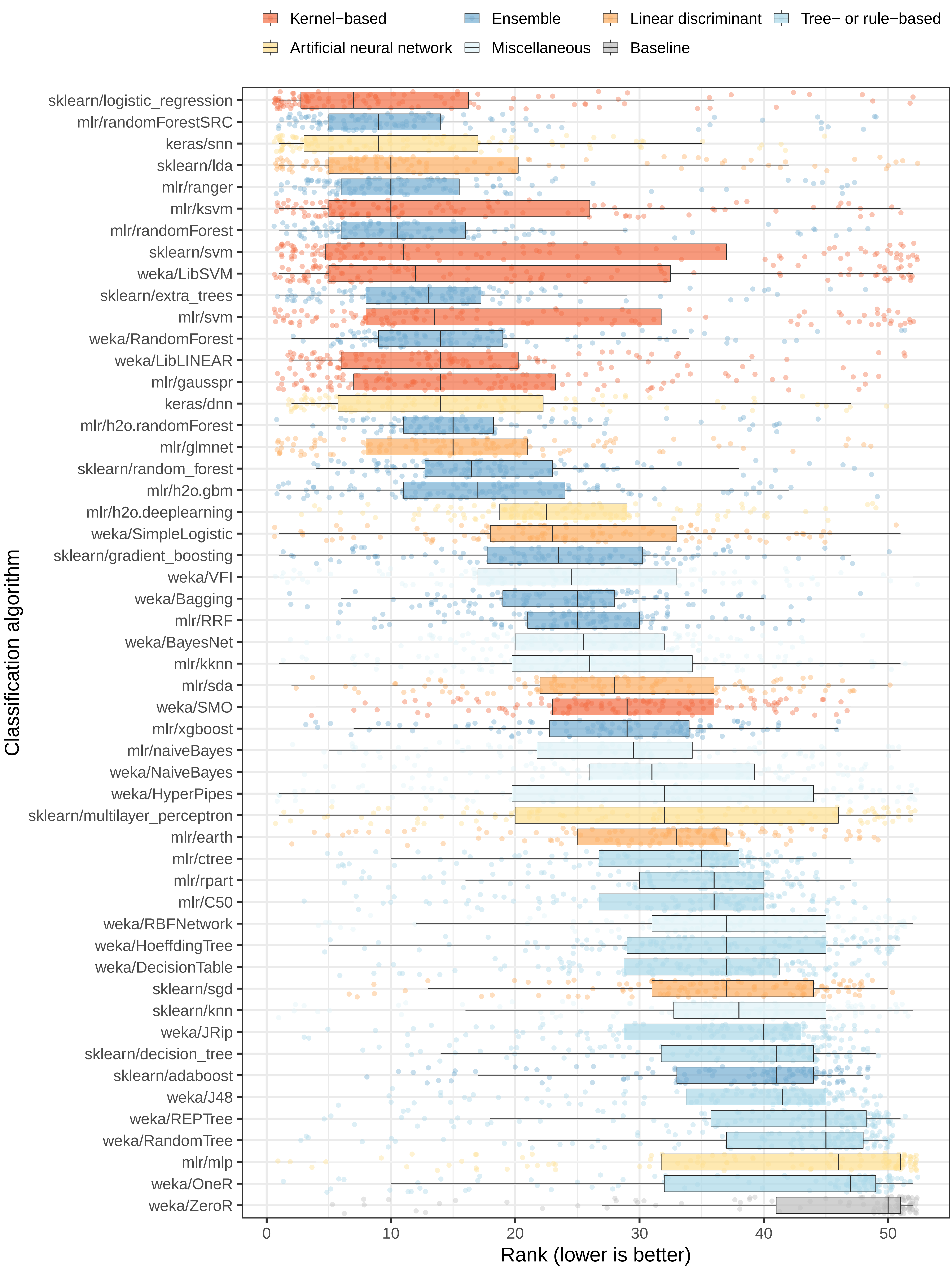

Kernel−based
Ensemble
Linear discriminant
Tree− or rule−based
Artificial neural network
Miscellaneous
Baseline
sklearn/logistic_regression
mlr/randomForestSRC
keras/snn
sklearn/lda
mlr/ranger
mlr/ksvm
mlr/randomForest
sklearn/svm
weka/LibSVM
sklearn/extra_trees
mlr/svm
weka/RandomForest
weka/LibLINEAR
mlr/gausspr
keras/dnn
mlr/h2o.randomForest
mlr/glmnet
sklearn/random_forest
mlr/h2o.gbm
mlr/h2o.deeplearning
weka/SimpleLogistic
sklearn/gradient_boosting
weka/VFI
weka/Bagging
mlr/RRF
weka/BayesNet
mlr/kknn
mlr/sda
weka/SMO
mlr/xgboost
mlr/naiveBayes
weka/NaiveBayes
weka/HyperPipes
sklearn/multilayer_perceptron
mlr/earth
mlr/ctree
mlr/rpart
mlr/C50
weka/RBFNetwork
weka/HoeffdingTree
weka/DecisionTable
sklearn/sgd
sklearn/knn
weka/JRip
sklearn/decision_tree
sklearn/adaboost
weka/J48
weka/REPTree
weka/RandomTree
mlr/mlp
weka/OneR
weka/ZeroR
Classification algorithm
0
10
20
30
40
50
Rank (lower is better)
