# Supplementary material for: The ability to classify patients based on gene-expression data varies by algorithm and performance metric
Source: PLoS Comput Biol. 2022 Mar 11;18(3):e1009926. doi: 10.1371/journal.pcbi.1009926 (PMC8942277; doi:10.1371/journal.pcbi.1009926)
Supplement: S14 Fig — We predicted patient states using gene-expression and clinical predictors with hyperparameter optimization (Analysis 4). We used nested cross validation to estimate which hyperparameter combination would be optimal for each algorithm in each training set. For each combination of dataset, class variable, and classification algorithm, we calculated the arithmetic mean of area under the receiver operating characteristic curve (AUROC) values across 5 iterations of Monte Carlo cross-validation. Next, we sorted the algorithms based on the average rank across all dataset/class combinations. Each data point that overlays the box plots represents a particular dataset/class combination. The algorithm rankings followed similar trends as Analysis 3 (no hyperparameter optimization); however, some differences are notable. For example, the weka/LibLINEAR and mlr/glmnet algorithms were ranked 11th and 16th in Analysis 3 (S13 Fig), but they were ranked 1st and 2nd in this analysis. (PDF) [file pcbi.1009926.s014.pdf]

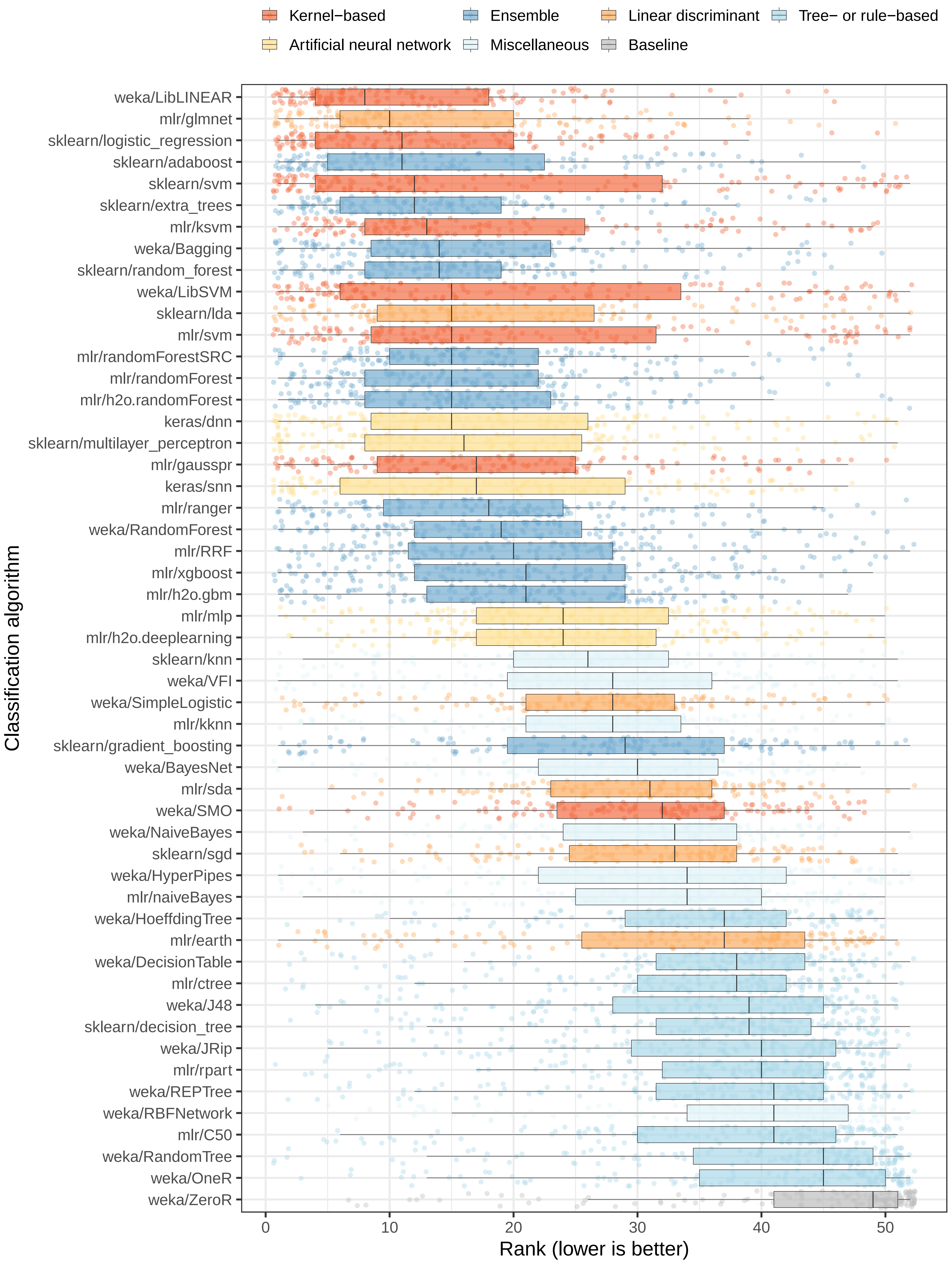

Kernel−based
Ensemble
Linear discriminant
Tree− or rule−based
Artificial neural network
Miscellaneous
Baseline
weka/LibLINEAR
mlr/glmnet
sklearn/logistic_regression
sklearn/adaboost
sklearn/svm
sklearn/extra_trees
mlr/ksvm
weka/Bagging
sklearn/random_forest
weka/LibSVM
sklearn/lda
mlr/svm
mlr/randomForestSRC
mlr/randomForest
mlr/h2o.randomForest
keras/dnn
sklearn/multilayer_perceptron
mlr/gausspr
keras/snn
mlr/ranger
weka/RandomForest
mlr/RRF
mlr/xgboost
mlr/h2o.gbm
mlr/mlp
mlr/h2o.deeplearning
sklearn/knn
weka/VFI
weka/SimpleLogistic
mlr/kknn
sklearn/gradient_boosting
weka/BayesNet
mlr/sda
weka/SMO
weka/NaiveBayes
sklearn/sgd
weka/HyperPipes
mlr/naiveBayes
weka/HoeffdingTree
mlr/earth
weka/DecisionTable
mlr/ctree
weka/J48
sklearn/decision_tree
weka/JRip
mlr/rpart
weka/REPTree
weka/RBFNetwork
mlr/C50
weka/RandomTree
weka/OneR
weka/ZeroR
Classification algorithm
0
10
20
30
40
50
Rank (lower is better)
