# Supplementary material for: The ability to classify patients based on gene-expression data varies by algorithm and performance metric
Source: PLoS Comput Biol. 2022 Mar 11;18(3):e1009926. doi: 10.1371/journal.pcbi.1009926 (PMC8942277; doi:10.1371/journal.pcbi.1009926)
Supplement: S18 Fig — In Analysis 4, we used nested cross validation to evaluate multiple hyperparameter combinations for each classification algorithm. We assessed the extent to which the area under the receiver operating characteristic curve (AUROC) varied across the hyperparameter combinations for each algorithm. For each combination of dataset, class variable, classification algorithm, and hyperparameter set, we averaged AUROC values across 5 Monte Carlo cross-validation iterations. Then we calculated the coefficient of variation for these averaged values across each combination of dataset/class and classification algorithm. Relatively low values indicate that the hyperparameter sets resulted in similar predictive performance. No results are available for 3 algorithms that used only a single hyperparameter option. (PDF) [file pcbi.1009926.s018.pdf]

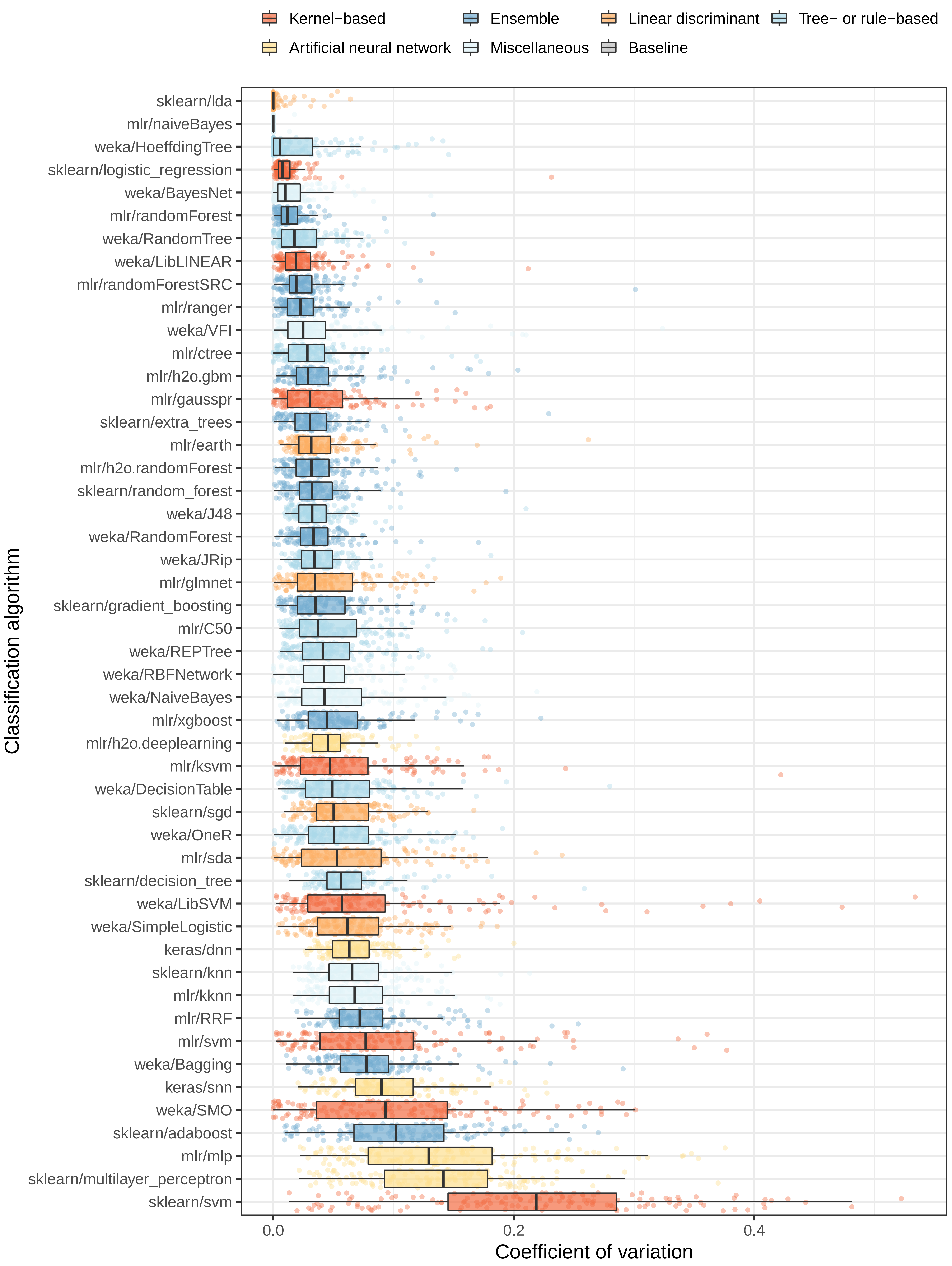

Kernel−based
Ensemble
Linear discriminant
Tree− or rule−based
Artificial neural network
Miscellaneous
Baseline
sklearn/lda
mlr/naiveBayes
weka/HoeffdingTree
sklearn/logistic_regression
weka/BayesNet
mlr/randomForest
weka/RandomTree
weka/LibLINEAR
mlr/randomForestSRC
mlr/ranger
weka/VFI
mlr/ctree
mlr/h2o.gbm
mlr/gausspr
sklearn/extra_trees
mlr/earth
mlr/h2o.randomForest
sklearn/random_forest
weka/J48
weka/RandomForest
weka/JRip
mlr/glmnet
sklearn/gradient_boosting
mlr/C50
weka/REPTree
weka/RBFNetwork
weka/NaiveBayes
mlr/xgboost
mlr/h2o.deeplearning
mlr/ksvm
weka/DecisionTable
sklearn/sgd
weka/OneR
mlr/sda
sklearn/decision_tree
weka/LibSVM
weka/SimpleLogistic
keras/dnn
sklearn/knn
mlr/kknn
mlr/RRF
mlr/svm
weka/Bagging
keras/snn
weka/SMO
sklearn/adaboost
mlr/mlp
sklearn/multilayer_perceptron
sklearn/svm
Classification algorithm
0.0
0.2
0.4
Coefficient of variation
