# Supplementary material for: The ability to classify patients based on gene-expression data varies by algorithm and performance metric
Source: PLoS Comput Biol. 2022 Mar 11;18(3):e1009926. doi: 10.1371/journal.pcbi.1009926 (PMC8942277; doi:10.1371/journal.pcbi.1009926)
Supplement: S24 Fig — For each class variable across all datasets, we assigned a category representing the type of patient state being predicted. For Analysis 5, we show the predictive performance for each combination of dataset, class variable, and classification algorithm in each class category. We use area under the receiver operating characteristic curve (AUROC) as a metric. The dashed, red line indicates the performance expected by random chance. The results are similar to those of Analyses 3 and 4 (S12 and S15 Figs). (PDF) [file pcbi.1009926.s024.pdf]

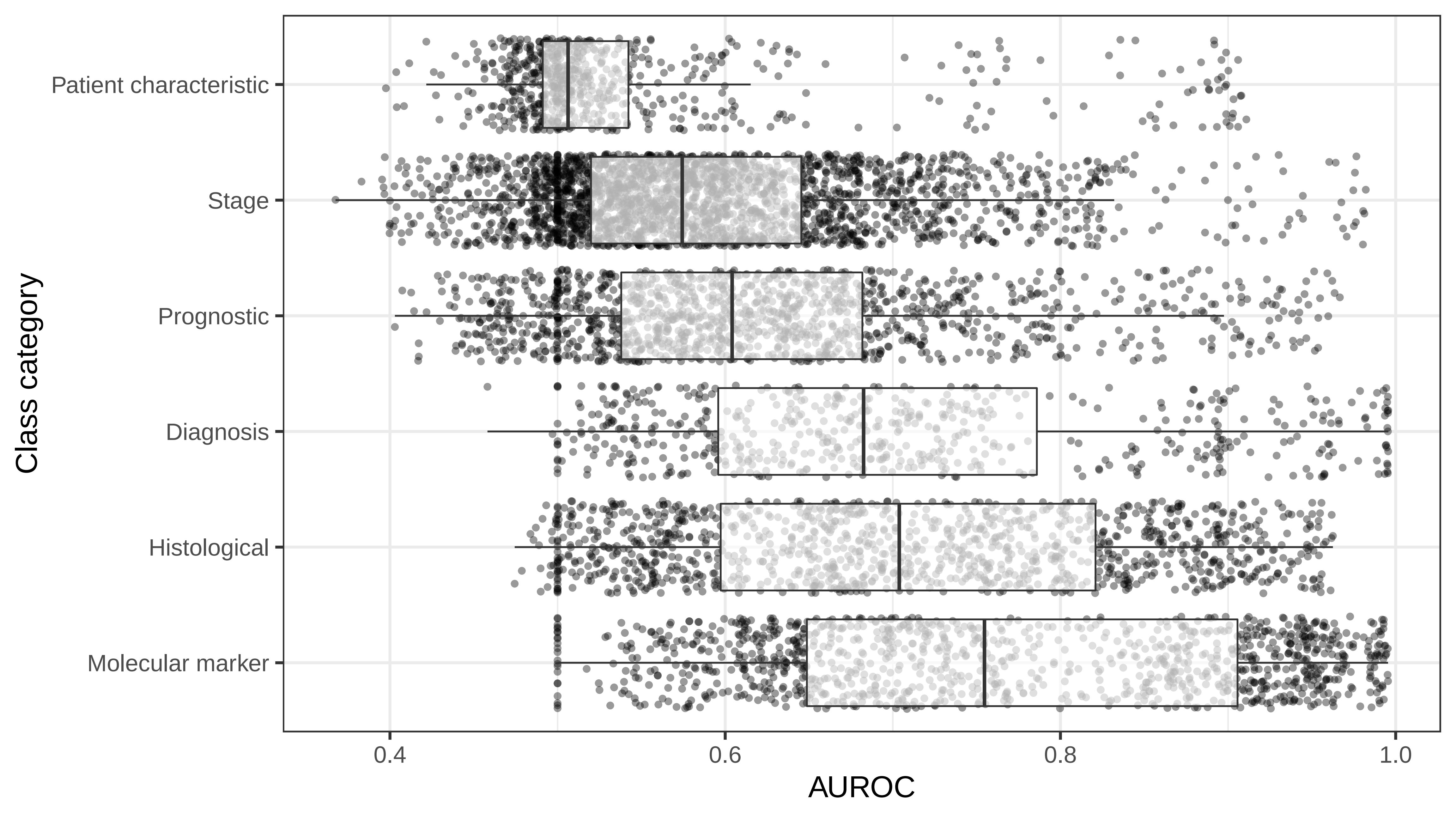
Patient characteristic
Stage
Prognostic
Diagnosis
Histological
Molecular marker
Class category
0.4
0.6
0.8
1.0
AUROC
