# Supplementary material for: The ability to classify patients based on gene-expression data varies by algorithm and performance metric
Source: PLoS Comput Biol. 2022 Mar 11;18(3):e1009926. doi: 10.1371/journal.pcbi.1009926 (PMC8942277; doi:10.1371/journal.pcbi.1009926)
Supplement: S31 Fig — We predicted patient states using gene-expression and clinical predictors with feature selection and optimization of the feature-selection algorithm hyperparameters (Analysis 6). We used nested cross validation to estimate which features and hyperparameter combinations would be optimal for each algorithm in each training set. (PDF) [file pcbi.1009926.s031.pdf]

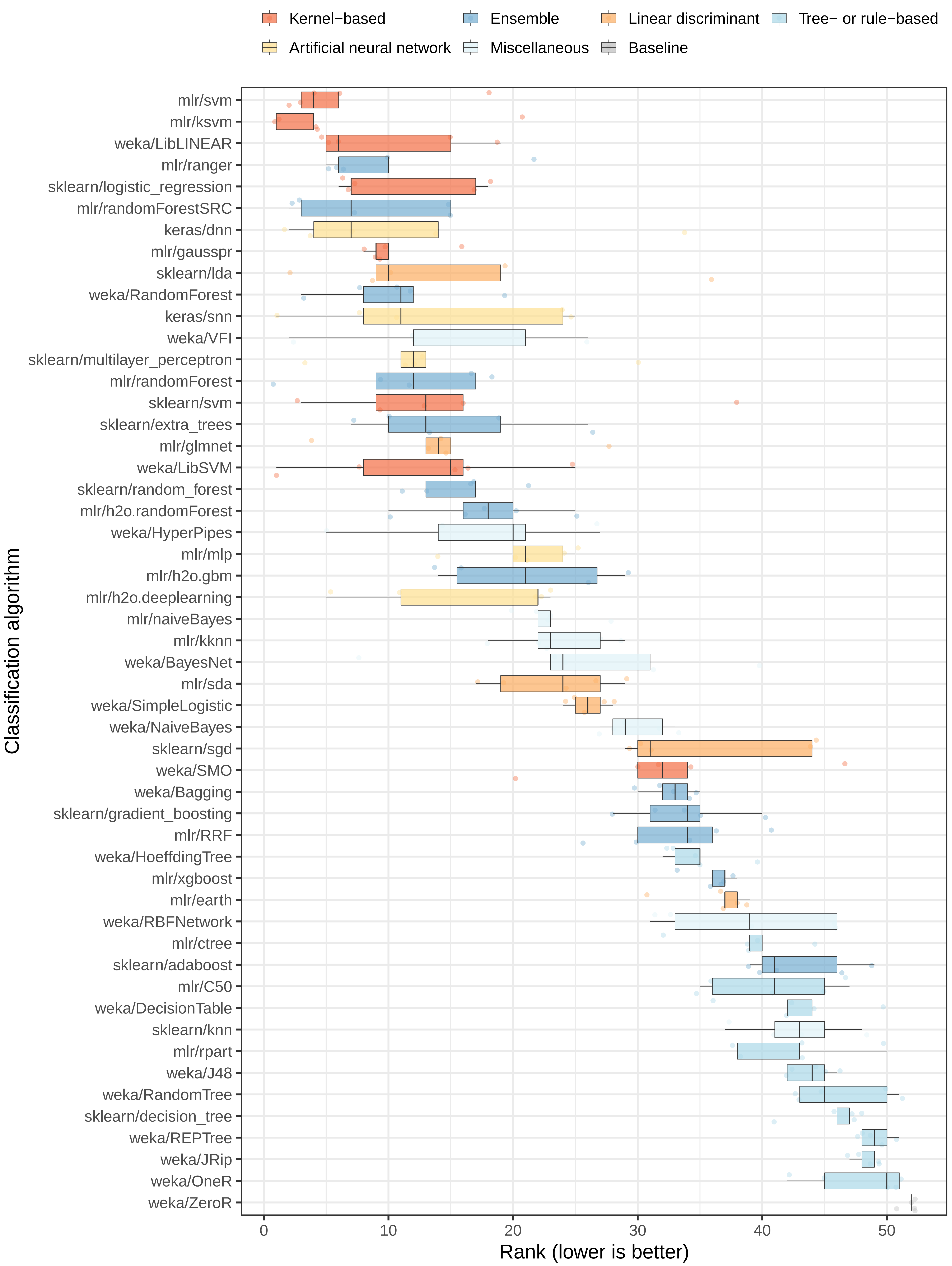

Kernel−based
Ensemble
Linear discriminant
Tree− or rule−based
Artificial neural network
Miscellaneous
Baseline
Classification algorithm
mlr/svm
mlr/ksvm
weka/LibLINEAR
mlr/ranger
sklearn/logistic_regression
mlr/randomForestSRC
keras/dnn
mlr/gausspr
sklearn/lda
weka/RandomForest
keras/snn
weka/VFI
sklearn/multilayer_perceptron
mlr/randomForest
sklearn/svm
sklearn/extra_trees
mlr/glmnet
weka/LibSVM
sklearn/random_forest
mlr/h2o.randomForest
weka/HyperPipes
mlr/mlp
mlr/h2o.gbm
mlr/h2o.deeplearning
mlr/naiveBayes
mlr/kknn
weka/BayesNet
mlr/sda
weka/SimpleLogistic
weka/NaiveBayes
sklearn/sgd
weka/SMO
weka/Bagging
sklearn/gradient_boosting
mlr/RRF
weka/HoeffdingTree
mlr/xgboost
mlr/earth
weka/RBFNetwork
mlr/ctree
sklearn/adaboost
mlr/C50
weka/DecisionTable
sklearn/knn
mlr/rpart
weka/J48
weka/RandomTree
sklearn/decision_tree
weka/REPTree
weka/JRip
weka/OneR
weka/ZeroR
0
10
20
30
40
50
Rank (lower is better)
