# Supplementary material for: The ability to classify patients based on gene-expression data varies by algorithm and performance metric
Source: PLoS Comput Biol. 2022 Mar 11;18(3):e1009926. doi: 10.1371/journal.pcbi.1009926 (PMC8942277; doi:10.1371/journal.pcbi.1009926)
Supplement: Attachment — Submitted filename: Response_to_Reviewers_PLOS_CompBio.pdf [file pcbi.1009926.s048.pdf]

DEPARTMENT OF BIOLOGY

Dear Editors:

Thank you for reviewing our manuscript entitled, "*Benchmarking 50 classification algorithms on 50 gene-expression datasets*." We have carefully reviewed the reviewers' comments and have addressed them in a point-by-point response, which can be found below. In addressing these comments, we performed hundreds of thousands of additional computing tasks, which took months to perform. Thank you for giving us time to complete those additional analyses, which we have added to the manuscript. Additionally, we have revised and restructured some of the text.

One of the reviewers suggested that we add neural-network algorithms to the analysis. Algorithms in this category have increased dramatically in usage within the computational-biology community. Thus we added two neural-network-based classification algorithms to our analysis, raising the number of classification algorithms to 52. Accordingly, it was necessary to change the title of our paper. However, rather than simply increase the number of classification algorithms in the title, we are proposing a new title that focuses more on the take-home message of the manuscript: "The ability to classify patients based on gene-expression data varies considerably across algorithms and performance metrics." If this is a problem, please let me know; we are flexible on this.

Warm regards,

Stephen R. Piccolo, PhD
Associate Professor
Department of Biology
Brigham Young University
(801) 422-7116
Stephen_Piccolo@byu.edu

We have listed the editor's and reviewers' comments in gray text below. Our comments are in bold, black text.

Reviewer #1, Line 344: Given the trend you have observed on the datasets you have used using clinical and/or gene expression data, please provide examples, if possible, of other algorithms that have not been studied but could potentially be promising, and why.

Thank you for taking the time to review the manuscript, and thank you for this suggestion. We have added 10 citations from diverse authors in this paragraph; we have cited examples of algorithms that were not included in this study but were designed specifically for feature selection and/or classification of gene-expression data. We did not include them because or focus was on general-purpose algorithms that have been implemented in open-source libraries.

BRIGHAM YOUNG UNIVERSITY – 4102 LSB – PROVO, UTAH 84602
(801) 422-2582 / FAX: (801) 422-0004

Reviewer #1, Line 356: Trying out convolutional neural networks in deep learning with optimizing number of layers and a hyperparameter search would be useful.

We have a three-part answer to this comment.

1. **In our original analysis, we included the H2O Deep Learning algorithm, which uses a "multi-layer feedforward artificial neural network that is trained with stochastic gradient descent using back-propagation." Although this algorithm is not a convolutional neural network, it is within the realm of deep learning.**
2. **Convolutional neural networks generally are used with data that have a spatial nature (e.g., images or DNA sequences) or that are structured as graphs, as highlighted in this paper, this paper, and this paper. Although it is possible that convolutional neural networks could be effective for classifying gene-expression data, work in this area would require algorithm-design efforts, which are outside the scope of this benchmark study.**
3. **We agree that deep neural networks are an important category of algorithms that can be used for classification. Thus, for this revision, we have added two more deep-learning algorithms, resulting in a total of 52 classification algorithms and 1116 hyperparameter combinations across these algorithms. The additional algorithms use implementations of deep neural networks from the keras package. For these algorithms, we have included an array of hyperparameter combinations that use diverse model types, layer architectures, regularization techniques, etc.**

Reviewer #1, Line 370: The remark about class imbalance being handled well by sklearn is interesting and valid.

Thank you for this comment. To emphasize this point, we have added another sentence that says, "Accordingly, future research that specifically focuses on subsampling, oversampling, and other methods to correct for class imbalance is warranted." Additionally, in response to one of the reviewer comments, we have calculated the area under the precision-recall curve for each set of results; this metric has been shown to be effective when classes are imbalance.

Reviewer #1, Line 377: It is interesting that data on co-occuring tumors did not have significance in feature selection.

Thank you for this comment.

Reviewer #2: Piccolo et al. benchmark 50 common classification algorithms from multiple publicly available packages to evaluate algorithm performance in a robust comparative framework. This is a very nice study. While much of their results are not particularly surprising (e.g., parameter optimization improves performance), I believe this study will be an important resource for a broad research community and one that is appropriate for publication in PLOS Computational Biology.

Thank you for your encouraging comments and for providing a careful review.

I have a few suggestions that I feel would improve the utility and breadth of audience for this work:

1) The AUROC analysis of this manuscript is great and will be a beneficial set of benchmarks for many studies. As the authors acknowledge, however, many studies have unbalanced classes that may see poor results compared to those expected from considering only auROC scores. To address this (and make their results more broadly applicable), it would be nice to see precision-recall curves in addition to their current analyses. The authors should have the data already to generate these plots, so I believe this should be a relatively easy addition.

Thank you for this suggestion. We have calculated auPRC scores for all parts of our analysis and have included these scores in the full set of results. We have added auPRC scores to our figure that shows a comparison across all of the metrics that we used. Additionally, we have created a figure that shows the correlation between auROC and auPRC scores. Furthermore, we added commentary about why auPRC may be more favorable in some circumstances than auROC. However, because the manuscript is already long and detailed, we chose not to include all of the same graphs for auPRC that we included for auROC. But an interested reader will be able to find these results in the supplementary data files we have posted online.

2) While I appreciate the author's focus on classification algorithms for classifying biomedical datasets, I believe there could be more attention given to other uses for classification algorithms. A discussion of classification algorithms as a discovery tool—such as using feature selection to identify potentially novel disease or phenotype-associated genes—would increase the breadth of their audience. Since the quality of feature selection is always dependent on the quality of the classification algorithm, but feature extraction is not equally accessible for all algorithms, this could lead to a very interesting additional contrast for the algorithms studied. The authors do touch on feature selection a bit, but mostly in reference to classification. It could be useful to have a brief discussion of how these algorithms perform for feature selection in a discovery context.

Thank you for this suggestion. We have updated the manuscript (Results section) to emphasize that feature selection (independent of classification) is a useful discovery tool. We have also added a simple "overlap" analysis using the Molecular Signatures Database where we have mapped top-ranked genes to disease-related, cellular signaling pathways as a way to illustrate one way to gain biological insight via feature selection.

3) I like that the authors contrast algorithm performance with running time. That said, I'm less certain that execution time should be valued as strongly as performance. Unless the difference is a matter of days or weeks, I suspect that pretty much all studies would choose the highest quality predictions over a modestly shorter runtime. Outside of (possibly) a few real-world clinical scenarios, I suspect the vast majority of studies would choose high quality predictions over even substantially longer runtimes.

Thank you for making this point. We have updated the text to emphasize that in most contexts, execution time is a less-critical factor than predictive performance. However, when the eventual goal of performing classification with gene-expression data is to provide useful tools for clinical applications, runtimes may be a more important consideration.

Minor comment: Figure 3 claims the y-axis is log10 transformed, but this does not seem to match the values along the axis.

Thank you. The reviewer is correct that we did not transform the values but rather transformed the coordinates on the y-axis (because some of the values are orders of magnitude larger than other

values). We have updated the caption for this figure to state that, "The coordinates for the y-axis have been transformed to a log-10 scale."

Reviewer #3: however, the great efforts of the author, the research is poorly organized. it's hard to get benefits for naive researcher?

We thank the reviewer for taking time to review the article. We are open to any particular feedback the reviewer may have on how to improve the organization of the article or how to make the article more accessible for naive researchers.

2. is svm-rfe multivariate? kindly check the type of feature selection methods

In the caption for Table 1, we indicate that we "assigned high-level categories that indicate whether the algorithms evaluate a single feature (univariate) or multiple features (multivariate) at a time." SVM-RFE uses the SVM algorithm to identify a decision boundary and then assign a weight to each feature. These weights are used to rank the features. Depending on the parameters used for this task, a single feature might be eliminated at a time. However, the manuscript states that we changed the parameters so that it would eliminate 5 features per iteration (to reduce computational time). Either way, the algorithm jointly evaluates all features when assigning the weights. This is why we described it as a multivariate method. An example of a univariate method, according to our way of describing the algorithms, is Information Gain, which calculates a score for each feature separately.

Reviewer #4: In this paper, the authors performed a benchmark comparison, applying 50 classification algorithms to 50 gene-expression datasets (143 class variables). The findings illustrate that algorithm performance varies considerably when other factors are held constant and thus that algorithm selection is a critical step in biomarker studies. The review paper may be useful for the researchers and students who are interested especially in the fields of characteristic genes of tumor.

Thank you for these comments and for taking time to provide a careful review.

However, a minor revision is required as indicated below:

1. The selection of tumor characteristic genes is a NP problem. Generally, feature selection algorithms can be divided into three categories: filter, wrapper and embedded. The wrapper method has the advantages of large search space coverage, more flexible classification accuracy and computational efficiency. Wrapper method uses meta heuristic algorithm to obtain the optimal feature subset, and combines the classification algorithm of machine learning as the evaluation standard, which achieves good results in feature selection of high-dimensional medical and health data. The authors should add the analysis of tumor gene feature selection using the meta heuristics.

Thank you for this comment. As the reviewer notes, our prior submission focused on filter-based and embedded feature selection. We have now added a section to our study where wrapper-based feature selection is used. However, this approach is extremely computationally intensive. In the limited time available to us to revise and resubmit, we had time to perform wrapper-based feature selection for two classification algorithms on five dataset/class combinations. Still, we have provided a framework that will enable this type of analysis to be advanced further in future studies.

2. It is suggested that the authors should simplify the introduction and make a more detailed analysis of the discussion.

Thank you for this suggestion. We have simplified the Introduction. We removed some of this content completely and integrated some of this content into the Discussion.

3. Traditional machine learning methods need to adjust super parameters in feature selection, so it is difficult to determine the best combination of parameters by the analytic method. So that the setting of optimal parameters itself is an optimization problem. Therefore, the parameter setting of the algorithm is worth exploring, and the author should give a detailed discussion.

We are not absolutely certain that we understood the reviewer's suggestion. If we understand correctly, the reviewer alludes to the fact that in Analysis 5, we used default hyperparameter combinations. The reviewer might also be referring to the difficulty, in general, of knowing which hyperparameters to tune and which options to specify. We have addressed both of these issues. We added a significant amount of analysis (requiring months of computer time) in which we performed feature selection for a variety of hyperparameter combinations and optimized across these options. Secondly, we have added brief commentary on the fact that it is a subjective decision to decide which hyperparameters to optimize.

4. It is suggested that the authors should provide the source programs of all 50 algorithms for better understanding and application of these methods.

We have expanded the "Data Availability Statement" to explain that our readers can find source code in three locations: 1) the code repositories for the software libraries that implemented each of the algorithms, 2) the code repository for the ShinyLearner tool, which we created for performing benchmarks, and 3) the code/data repository for this specific analysis.

Have the authors made all data and (if applicable) computational code underlying the findings in their manuscript fully available? The PLOS Data policy requires authors to make all data and code underlying the findings described in their manuscript fully available without restriction, with rare exception (please refer to the Data Availability Statement in the manuscript PDF file). The data and code should be provided as part of the manuscript or its supporting information, or deposited to a public repository. For example, in addition to summary statistics, the data points behind means, medians and variance measures should be available. If there are restrictions on publicly sharing data or code —e.g. participant privacy or use of data from a third party—those must be specified.

Reviewer #1: Yes

Reviewer #2: Yes

Reviewer #3: None

Reviewer #4: Yes

Thank you.

PLOS authors have the option to publish the peer review history of their article (what does this mean?). If published, this will include your full peer review and any attached files.

If you choose "no", your identity will remain anonymous but your review may still be made public.

Do you want your identity to be public for this peer review? For information about this choice, including consent withdrawal, please see our Privacy Policy.

Reviewer #1: No

Reviewer #2: No

Reviewer #3: Yes: muhammed abd-elnaby sadek

Reviewer #4: No

Thank you.

Figure Files:

While revising your submission, please upload your figure files to the Preflight Analysis and Conversion Engine (PACE) digital diagnostic tool, https://pacev2.apexcovantage.com. PACE helps ensure that figures meet PLOS requirements. To use PACE, you must first register as a user. Then, login and navigate to the UPLOAD tab, where you will find detailed instructions on how to use the tool. If you encounter any issues or have any questions when using PACE, please email us at figures@plos.org.

We have done this for all of the figures from the main part of the manuscript and uploaded them as TIF files.

Data Requirements:

Please note that, as a condition of publication, PLOS' data policy requires that you make available all data used to draw the conclusions outlined in your manuscript. Data must be deposited in an appropriate repository, included within the body of the manuscript, or uploaded as supporting information. This includes all numerical values that were used to generate graphs, histograms etc.. For an example in PLOS Biology see here:
http://www.plosbiology.org/article/info%3Adoi%2F10.1371%2Fjournal.pbio.1001908#s5.

All data used in the analysis and used to generate figures have been deposited in a repository on the Open Science Framework (https://osf.io/fv8td/) and are available without restriction.

Reproducibility:

To enhance the reproducibility of your results, we recommend that you deposit your laboratory protocols in protocols.io, where a protocol can be assigned its own identifier (DOI) such that it can be cited independently in the future. Additionally, PLOS ONE offers an option to publish peer-reviewed clinical study protocols. Read more information on sharing protocols at

https://plos.org/protocols?utm_medium=editorial-email&utm_source=authorletters&utm_campaign=pr
otocols
