# Supplementary material for: The ability to classify patients based on gene-expression data varies by algorithm and performance metric
Source: PLoS Comput Biol. 2022 Mar 11;18(3):e1009926. doi: 10.1371/journal.pcbi.1009926 (PMC8942277; doi:10.1371/journal.pcbi.1009926)
Supplement: Attachment — Submitted filename: reviewer_ploscompio_june2021.docx [file pcbi.1009926.s046.docx]

Line 344: Given the trend you have observed on the datasets you have used using clinical and/or gene expression data, please provide examples, if possible, of other algorithms that have not been studied but could potentially be promising, and why.

Line 356: Trying out convolutional neural networks in deep learning with optimizing number of layers and a hyperparameter search would be useful.

Line 370: The remark about class imbalance being handled well by sklearn is interesting and valid.

Line 377: It is interesting that data on co-occuring tumors did not have significance in feature selection.
