# Supplementary material for: The ability to classify patients based on gene-expression data varies by algorithm and performance metric
Source: PLoS Comput Biol. 2022 Mar 11;18(3):e1009926. doi: 10.1371/journal.pcbi.1009926 (PMC8942277; doi:10.1371/journal.pcbi.1009926)
Supplement: Attachment — Submitted filename: New Rich Text Document.rtf [file pcbi.1009926.s047.rtf]

Benchmarking 50 classification algorithms on 50 gene-1expression datasets

comments :
however, the great efforts of the author, the research is poorly organized. it's hard to get benefits for naive researcher?
2. is svm-rfe multivariate? kindly check the type of feature selection methodss
